# CNNKSCEC: a deep learning-based framework for chromatin loop prediction with multi-source feature integration

**DOI:** 10.3389/fgene.2026.1850219

**Published:** 2026-07-03

**Authors:** Junfeng Wang, Bingzi Zheng, Lili Wu, Xiaoyan Liu, Haixia Zhai, Junwei Luo

**Affiliations:** School of Software, Henan Polytechnic University, Jiaozuo, China

**Keywords:** bioinformatics, chromatin loops, deep learning, DNase, Hi-C, feature integration

## Abstract

**Motivation:**

Chromatin in the cell nucleus adopts a complex three-dimensional (3D) structure shaped by folding and interactions, with chromatin loops serving as fundamental organizational units. Accurate loop prediction is essential for understanding gene regulation and disease mechanisms. However, existing chromatin loop prediction methods still face challenges in noise handling, data imbalance, and multi-omics integration.

**Results:**

In this study, we present CNNKSCEC, a deep learning-based framework for chromatin loop prediction via multi-source feature fusion. The model integrates Hi-C and DNase-seq data into a dual-channel feature matrix as input. It employs a three-stage iterative feature extraction framework consisting of a dual-branch convolutional module (CNNC), a SCConv module combining SRU and CRU, and an ECHybridAddition module integrating both ECA and CBAM attention mechanisms. This design enables iterative multi-scale feature extraction and enhances the feature representation capability of the input matrix. Finally, the model uses a fully connected layer for classification, generating candidate chromatin loops with prediction scores, and filters out false candidates through density-based clustering. In the experiments, we compare CNNKSCEC with existing chromatin loop prediction methods, and the results demonstrate that the approach outperforms other methods overall in terms of performance. The code is available from https://github.com/zhengbingzi/CNNKSCEC.git.

## 1 Introduction

The cell nucleus serves as the storage site for genetic material in eukaryotic cells, and chromatin being the primary form in which genetic material exists within the nucleus. Within the nucleus, chromatin is not arranged linearly but adopts a complex three-dimensional (3D) architecture through folding and interactions. These interactions, arising from the intricate folding, play critical roles in gene transcription, directly influencing gene expression regulation and cellular functions (Rao et al., 2014). Therefore, investigating the 3D genome organization is of paramount importance. Over the past decades, numerous methods have been developed to study the structural complexity of the 3D genome, many of which are based on the concept of Chromosome Conformation Capture (3C) (Stephenson-Gussinye and Furlan-Magaril, 2023). With the continuous advancement of 3C technologies and bioinformatics, researchers have been deepening their research on three-dimensional genome organization. From the perspective of whole genome, the multi-scale high-dimensional chromatin structures are classified into chromosome territory (CT), A/B compartment, topologically associated domain (TAD) and chromatin loop according to their hierarchical structure from large to small (Dekker et al., 2013; Gorkin et al., 2014; R et al., 2015; Wang S. et al., 2022).

Chromatin loops are fundamental units of the 3D chromatin architecture, characterized by spatially proximal interactions between chromatin segments that are linearly distant along the genome. Recent studies have revealed that loop formation primarily depends on the CCCTC-binding factor (CTCF) and the cohesin complex through a “loop extrusion” mechanism (Sanborn et al., 2015; Fudenberg et al., 2016). Increasing evidence indicates that genetic or structural variations at loop anchors are often associated with target genes and linked to developmental disorders and diseases (Mumbach et al., 2017; Liu et al., 2023). Therefore, accurate identification of chromatin loops is of critical importance.

With continued exploration and technological advances, an increasing number of methods have been developed for detecting chromatin loops. Currently, the most common approaches for chromatin loop prediction are based on Hi-C sequencing. Hi-C (Lieberman-Aiden et al., 2009; Dixon et al., 2012), as a high-throughput chromosome conformation capture technique, enables comprehensive mapping of the genome’s three-dimensional organization by detecting spatial interactions between chromatin fragments. In the early stages of Hi-C data analysis, traditional analytical methods based on statistics and image processing dominated chromatin loop prediction studies. For example, HiCCUPS (Rao et al., 2014; Neva and Durand, 2016) identifies candidate chromatin loops by comparing the Hi-C contact count of each pixel against multiple local backgrounds, combined with Poisson significance testing and Benjamini–Hochberg FDR correction, selecting local maxima that are significantly higher than the background. Fit-Hi-C (Ay et al., 2014), on the other hand, assigns statistical confidence estimates to mid-range intrachromosomal contacts by jointly modeling random polymer loop effects and technical biases observed in previous Hi-C datasets. SIP (Rowley et al., 2020) treats Hi-C contact maps as two-dimensional images, identifies candidate loops through image enhancement and local extrema detection, and improves detection reliability by incorporating statistical filtering steps such as Poisson testing and empirical FDR estimation. With the growing application of machine learning and deep learning, new strategies for chromatin loop prediction have emerged. For example, Peakachu (Salameh et al., 2020) is a supervised learning-based Hi-C chromatin loop detection method that predicts potential loop structures from whole-genome contact maps by training a random forest model with positive and negative samples generated from known loops, thereby enhancing the sensitivity and accuracy of detection. DeepLoop (Zhang et al., 2022) is a deep learning-based Hi-C analysis tool that addresses noise and sparsity in low-coverage data through a dual-module design combining HiCorr bias correction and U-Net signal enhancement. GILoop (Wang F. et al., 2022) is a deep learning-based chromatin loop detection method that integrates image and graph-structured representations. By jointly learning Hi-C contact matrix features through a dual-branch network composed of U-Net and GCN, GILoop improves the prediction performance of CTCF-mediated chromatin loops under low sequencing depth and across different cell lines. In addition, researchers have applied epigenomic data such as ChIP-seq (Johnson et al., 2007), ChIA-PET (Paulsen et al., 2014), and DNase-seq (Song and Crawford, 2010) to chromatin loop prediction, leading to the development of methods that leverage epigenomic features for more comprehensive and accurate identification of chromatin loops and for exploring their biological functions. ChIA-PET (Fullwood et al., 2009) enables high-resolution detection of protein-mediated chromatin loops across the genome, while ChIA-PET2 (Li et al., 2017) integrates all analytical steps required for ChIA-PET data processing, allowing analysis of different types of ChIA-PET datasets from raw sequencing reads to loop identification. CLoops (Yaqiang et al., 2020) is a two-step algorithm based on DBSCAN and PLB, which utilizes 3D epigenomic datasets such as ChIA-PET and HiChIP (Paulsen et al., 2014; Mumbach et al., 2016) to identify significant loops through clustering and statistical testing, thereby improving the accuracy of chromatin loop detection.

Although these methods have achieved certain advances, several challenges remain, such as how to extract multiscale features from contact maps, how to fully leverage the biological information in the data, and how to improve the accuracy of chromatin loop prediction. To address these issues, we propose CNNKSCEC, a more robust and extensible deep learning-based framework for feature representation and integration that builts upon existing chromatin loop detection methods. First, spatially aligned Hi-C and DNase-seq (Liu et al., 2019) data are integrated into a dual-channel feature fusion matrix, preserving local interactions and chromatin accessibility patterns. Next, a multi-branch feature extraction architecture combined with a feature-level self-distillation is employed. Specifically, three iterations of dual-branch convolutional modules (CNNC, a feature extraction module consisting of two parallel CNN branches), the SCConv (Li et al., 2023) module-which integrates the Spatial Reconstruction Unit (SRU) and Channel Reconstruction Unit (CRU), and the ECHybridAddition module, which combines the lightweight Efficient Channel Attention (ECA) (Wang et al., 2020) and Convolutional Block Attention Module (CBAM) (Bahdanau et al., 2014; Woo et al., 2018) are used to iteratively extract multiscale features and enhance the representation of the feature matrix. Moreover, we employ KL divergence as the distillation loss within the feature-level self-distillation framework to align the probability distribution of student features with that of teacher features (Hinton et al., 2015; Habib et al., 2024). Finally, CNNKSCEC generates candidate chromatin loops with associated prediction scores, and filters those with scores below 0.97 through density clustering. In the experimental evaluation, the predicted loops were subjected to a series of validation analyses and compared with other chromatin loop prediction methods, demonstrating that CNNKSCEC achieves superior performance.

## 2 Methods

CNNKSCEC is a deep learning method for detecting chromatin loops in the 3D genome. It takes Hi-C and DNase feature matrices as input, transforming the chromatin loop prediction into a binary classification problem. The CNNKSCEC prediction process is divided into five steps:(i) Data Preprocessing: Submatrices of size 21 × 21 are generated. (ii) Feature Extraction: CNNKSCEC uses CNNC, SCConv, ECHybridAddition and Self-distillation for multi-scale feature extraction. (iii) Prediction: The model outputs the prediction results for candidate chromatin loops in the form of probabilities through a fully connected layer. (iv) Clustering: The predicted candidate chromatin loops are clustered based on density to obtain the final chromatin loop predictions.

**FIGURE 1:**
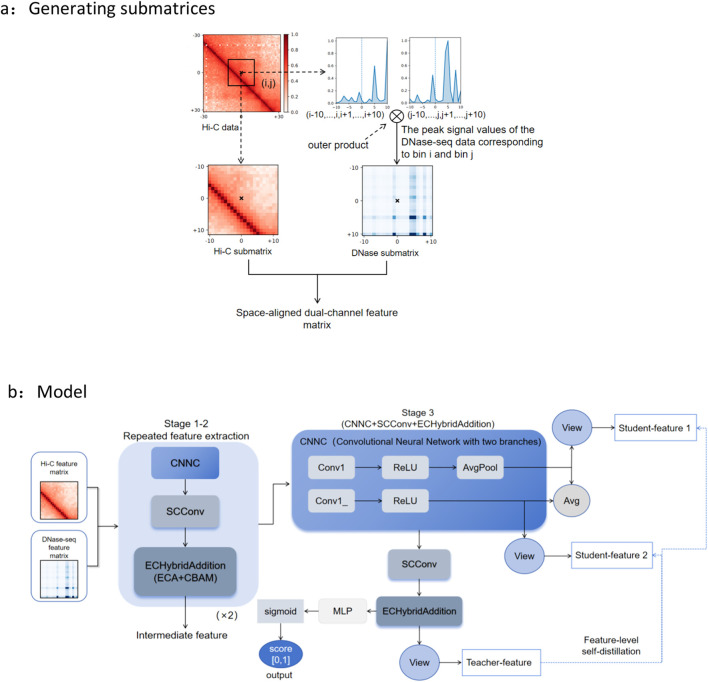
CNNKSCEC framework diagram.**(a)** Generation of submatrices for dual-channel spatial alignment. **(b)** The workflow of CNNSCEC.

The workflow of CNNKSCEC is shown in Figure 1.

### 2.1 Data pre-processing

#### 2.1.1 Construction of Hi-C feature matrix

Each chromosome was divided into subregions of equal length (bins) at a resolution of res (5 kb by default). Using juicer_tools, the interaction matrix for each chromosome at the specified resolution was obtained. To remove systematic biases in the Hi-C data, the raw interaction matrices were normalized using the KR method. It is generally recognized that chromatin loop anchors exhibit strong interactions, whereas interactions that are too short or too long may hinder the formation of the loop structure. Therefore, the genomic distance threshold was set at 30 kb to 3 Mb in this study.

For one chromosome, a Hi-C interaction matrix M was obstained, where M[i,j] represents the contact frequency between the ith bin and the jth bin. For M[i,j], if it meets the following condition, we produce a sub-matrix SM_ij_, where the center point B_ij_ of SM_ij_ satisfy the formula 1:

$$B_{ij}=\left[i,j\right],where\;M\left[i,j\right]>1\;and\frac{lower}{res}\leq i-j\leq \frac{upper}{res}$$ (1)

where *res* represents the resolution, *lower* indicates the minimum distance between anchor points (default value is 30 kb), and *upper* indicates the maximum distance between anchor points (default value is 3 Mb).

Next, we define d as half the side length of the sub-matrix. Then, the sub-matrix *SM*_*ij*_ = M[i-d:i + d+1, j-d:j + d+1], which indicates the elements in M from the (i-d)th row to the (i + d)th row and from the (j-d)th column to the (j + d)th column, where the number of rows and columns of the sub-matrix are both 2 d + 1. In this study, we set d = 10 to generate sub-matrices of size 21 × 21.

#### 2.1.2 Construction of DNase feature matrix

Map *B*_*ij*_ to the corresponding chromatin fragments on the chromosome. For each obtained pair of chromatin fragments, compute the average DNase-seq signal values in res kb bins. For each fragment, extend d bins on both sides to construct a chromatin fragment neighborhood of 2d + 1 bins in length. Taking the averaged signal values as vector entries, each pair of interacting chromatin fragments corresponds to two 1 × 21 vectors, denoted as X = {X_1_,X_2_,X_n-1_,X_n_} (with the ith bin as the center) and Y = {Y_1_,Y_2_,Y_n-1_,Y_n_} (with the jth bin as the center). Then transpose Y and compute the outer product with X to obtain the DNase feature matrix SD_ij_.

#### 2.1.3 Merge the Hi-C and DNase feature matrices

For each B_ij_, we merge the corresponding SM_ij_ and SD_ij_ into a new matrix AM_ij_.

### 2.2 Feature extraction module

This method designs an efficient multi-branch feature extraction framework, CNNKSCEC, by integrating CNNC, SCConv (combine SRU and CRU), ECHybridAddition (combine ECA and CBAM) and Self-distillation. Specifically, the CNNC module consists of a dual-branch convolutional network, while the SCConv module not only improve the network’s expressive capability but also mitigate overfitting during training through regularization and gating mechanisms. The final prediction is obtained via a fully connected layer. The multi-branch convolutional architecture of this method effectively improves the model’s generalization and stability.

In this method, the input feature matrix is first fed into the CNNC layer for preliminary feature extraction. The feature matrix output by the CNNC layer is then passed to the SCConv module to further capture local and spatial contextual information. To achieve feature reuse and residual information fusion, the feature matrix processed by the SCConv module is added to the corresponding feature matrix from the previous layer. The fused feature matrix is subsequently fed into the ECHybridAddition module for further feature integration and recalibration. To fully extract and fuse multi-level features, the above process is repeated three times. Finally, the resulting feature representation is fed into a fully connected layer for feature aggregation, and the final prediction result is generated through a sigmoid function.

#### 2.2.1 CNNC module

The CNNC module adopts a dual-branch convolutional architecture, and its input is a two-channel feature matrix composed of spatially aligned Hi-C and DNase-seq features. To extract initial spatial features at different scales, the input feature matrix is simultaneously fed into the upper and lower convolutional branches in the first iteration. Specifically, the upper branch uses a 5 × 5 convolutional kernel to enlarge the initial receptive field and capture contextual information within a broader neighborhood, while the lower branch uses a 3 × 3 convolutional kernel to preserve finer local interaction features.

In subsequent iterations, since the feature maps have already been processed by the previous convolution and fusion operations and contain certain contextual information, both branches use 3 × 3 convolutional kernels. This setting further refines local features while reducing the number of parameters and computational cost, and avoids excessive feature smoothing caused by repeatedly using larger convolutional kernels.

In each iteration, the convolutional outputs of both branches are activated by ReLU. Then, the output of the upper branch is further processed by average pooling to smooth feature responses and enhance the robust representation of contextual information, whereas the lower branch does not use pooling in order to preserve fine-grained local spatial information as much as possible. Finally, the output of the CNNC module is obtained by averaging the outputs of the two branches, defined as formula 2:

$$o\text{ut}=\frac{out_{1}+out_{1_{-}}}{2}$$ (2)

where *out*_1_ and *out*_1__ denote the outputs of the upper and lower branches, respectively.

#### 2.2.2 SCConv module

The SCConv module combines a Spatial Reconstruction Unit (SRU) and a Channel Reconstruction Unit (CRU) to enhance feature representation. Specifically, SRU performs spatial reconstruction, while CRU refines channel-wise dependencies. This design improves robustness to complex data and reduces computational cost. The overall operation is defined as formula 3:

$$X_{out}=CRU\left(SRU\left(X\right)\right)$$ (3)

where *X* denotes the input features, *X*_*out*_ is the output, *SRU*(·) represents the spatial reconstruction operation, and *CRU*(·) denotes the channel reconstruction operation.

##### 2.2.2.1 SRU

The SRU combines group normalization with a gating mechanism to enhance the feature representation capability of the model. The core of this module is to compute adaptive weight coefficients to regulate feature responses, thereby effectively suppressing noisy and redundant features while highlighting key information related to chromatin loop identification. Specifically, the SRU divides the input features into high-information and low-information parts through a gating operation. These two parts are then re-fused through feature reconstruction at the output stage, which enhances the network’s ability to learn Hi-C contact patterns and DNase-seq chromatin accessibility features. The output of the SRU is defined by formulas 4-6:

$$w_{1}=\{\begin{array}{cc} 1, & \text{if }\sigma \left(\text{GN}\left(x\right)\cdot w\gamma \right)>\text{Threshold}\\ \sigma \left(\text{GN}\left(x\right)\cdot w\gamma \right), & \text{otherwise} \end{array}$$ (4)

$$w_{2}=\{\begin{array}{cc} 0, & \text{if }\sigma \left(\text{GN}\left(x\right)\cdot w\gamma \right)>\text{Threshold}\\ \sigma \left(\text{GN}\left(x\right)\cdot w\gamma \right), & \text{otherwise} \end{array}$$ (5)

$$y=\text{RS}\left(\text{Split}\left(w_{1}⊗x\right),\text{Split}\left(w_{2}⊗x\right)\right)$$ (6)

where *x* and *y* denote the input and output, *GN*(·) is group normalization, *σ* is the sigmoid function, *Threshold* is the gating parameter, *Split* is feature partitioning, and *RS*(·) is feature reconstruction.

##### 2.2.2.1 CRU

CRU enhances the network’s ability to handle complex data structures through a channel-splitting and reconstruction mechanism. It employs adaptive channel reorganization by dividing the input into high- and low-channel features. The high-channel branch is processed with group convolution (GWC) and pointwise convolution (PWC), while the low-channel branch combines PWC with the original features. The two branches are then concatenated and compressed using adaptive pooling to obtain compact representations. The operation is defined as formula 7:

$$output=Pool\left(Concat\left[\left(GWC\left(x_{up}\right)+PWC\left(x_{up}\right)\right),Concat\left[PWC\left(x_{low}\right),x_{low}\right]\right]\right)$$ (7)

where *output* denotes the feature output, *x*_*up*_ and *x*_*low*_ are the high- and low-channel inputs, *GWC*(·) and *PWC*(·) represent group and pointwise convolutions, *Concat* is concatenation, and *Pool* denotes adaptive pooling.

#### 2.2.3 ECHybridAddition module

The ECHybridAddition module enhances feature discrimination by applying attention to feature maps, which often contain redundant information. It combines ECA for efficient channel-wise modeling and CBAM for sequential channel and spatial attention, allowing the network to highlight salient features with low computational cost. The operation is defined as formula 8:

$$Y_{out}=CBAM\left(ECA\left(Y\right)\right)$$ (8)

where *Y* is the input feature, *ECA*(·) performs lightweight channel modeling, and *CBAM*(·) applies combined channel and spatial attention.

##### 2.2.3.1 ECA

ECA models inter-channel dependencies efficiently using a 1D convolution instead of fully connected layers. It first applies global average pooling (GAP) to obtain a channel descriptor, then captures correlations between neighboring channels through local convolution, and finally generates per-channel weights via a sigmoid function. The operation is defined as formula 9:

$$Y_{ECA}=Y⊗\sigma \left(Conv1D\left(GAP\left(Y\right)\right)\right)$$ (9)

where *Y* denotes the input feature, *Y*_*ECA*_ is the output after ECA processing, and *GAP* refers to global average pooling.

##### 2.2.3.2 CBAM

CBAM applies attention sequentially along channel and spatial dimensions to refine features. Channel attention highlights important channels via global pooling and a shared MLP, while spatial attention focuses on salient regions using pooled channel information and convolution. The operation is defined as formula 10:

$$Y_{CBAM}=Y_{ECA}⊗M_{c}\left(Y_{ECA}\right)⊗M_{s}\left(Y_{ECA}⊗M_{c}\left(Y_{ECA}\right)\right)$$ (10)

where *Y*_*ECA*_ is the input feature, *M*_*c*_(·) is channel attention, and *M*_*s*_(·) is spatial attention.

#### 2.2.4 Self-distillation module

Knowledge distillation is commonly used to transfer information from a teacher model to a student model. In this study, we adopt a feature-level self-distillation strategy within the same feature extraction framework. The teacher and student branches share the shallow feature extraction process but differ in the depth of subsequent feature transformation.

Specifically, the teacher branch follows the complete three-stage feature extraction pipeline. Each stage consists of CNNC, SCConv, and ECHybridAddition modules. In contrast, the student branch adopts a lighter structure. Its first two stages are the same as those of the teacher branch, while only the CNNC module is retained in the third stage. The student features are extracted after the third CNNC module and are then flattened for subsequent prediction and distillation training. Therefore, compared with the teacher branch, the student branch does not pass through the third-stage SCConv and ECHybridAddition modules.

During training, features generated by the subsequent modules in the teacher branch are used to provide auxiliary guidance for the student features extracted from the third CNNC module. The supervised classification loss is calculated between the student prediction and the ground-truth label. In addition, a distillation loss is introduced to constrain the difference between the teacher and student feature distributions. Specifically, the feature representations of the teacher and student branches are converted into probability distributions using the softmax function, and KL divergence is used to measure their distributional discrepancy.

### 2.3 Model prediction

The CNNKSCEC model was used to perform predictions on chromosomes 20–22. During the prediction phase, the model loads the best weights obtained during training and performs a forward pass to compute the model’s output and to generate predictions. As a binary classification task, the model outputs a continuous probability value between 0 and 1, representing the confidence of chromatin loop formation. Finally, a density-based clustering algorithm, as used in Peakachu, is applied to the predicted chromatin loops for further filtering. The clustering threshold is set to 0.97 to obtain the final predicted chromatin loops. It is important to emphasize that the discriminative power of CNNKSCEC derives from its deep feature learning architecture, which operates on spatially aligned Hi-C and DNase fusion matrices. The density-based clustering step does not introduce additional discriminative information; rather, it serves to consolidate high-confidence interaction signals, improve structural consistency, and reduce redundant local predictions.

### 2.4 Model training

#### 2.4.1 Construction of positive and negative samples

##### 2.4.1.1 Positive sample centers and matrices

We used ChIA-PET and HiChIP data to define positive and negative samples. Specifically, genomic regions from H3K27ac HiChIP (Ying et al., 2020) and CTCF ChIA-PET (Paulsen et al., 2014) were mapped onto res-kb bins. Redundant interactions were removed by excluding H3K27ac pairs contained within CTCF interactions and vice versa. The processed datasets were then merged to generate a BEDPE file containing chromatin interaction coordinates and chromosome identifiers. Multi-interval interactions were further split into single intervals, and the final dataset was grouped by chromosome to produce positive sample center files. Each center is used to extract 21 × 21 submatrices from Hi-C and DNase-seq data. Hi-C and DNase submatrices are then combined to form the final positive sample feature matrix.

##### 2.4.1.2 Negative sample centers and matrices

Negative sample centers are generated based on positive sample centers. For each positive center, negative centers are selected from non-positive positions within the Hi-C matrix according to three criteria: i) Select twice the number of pairs at a distance equal to that of the corresponding positive interaction between the two chromatin segments; ii) Select an equal number of pairs at a distance greater than the maximum distance of the corresponding positive interaction; iii) Randomly select an equal number of pairs at any distance. For each chromosome, negative samples are filtered and deduplicated according to the above criteria, resulting in roughly a 1:2 ratio of positive to negative interaction center points.

Negative sample matrices are constructed using the same procedure as for positive samples.

#### 2.4.2 Model training

The model is trained within a standard supervised learning framework, iteratively optimizing parameters over multiple epochs. Each epoch consists of a training and a validation phase. During training, intermediate features are computed via forward propagation, and the total loss is calculated by combining task loss (cross-entropy) and distillation loss (KL divergence). Model parameters are updated through backpropagation and an optimizer. In the validation phase, performance on the validation set is evaluated, and the model achieving the highest F1-score is saved as the best model for subsequent prediction tasks.

##### 2.4.2.1 Construction of training and validation samples

CNNKSCEC uses chromosomes 1–19 from the GM12878 cell line for training and validation. Positive and negative samples are combined, shuffled, and split 4:1 into training and validation sets. Training batches are shuffled each epoch; validation data remain ordered for evaluation.

##### 2.4.2.2 Construction of test samples

For testing, CNNKSCEC uses chromosomes 20–22 from the GM12878 cell line. Positive and negative samples from these chromosomes are combined into a unified dataset for evaluation.

##### 2.4.2.3 Loss function

This study uses a dual-objective loss combining supervised learning and feature distillation. BCE ensures classification accuracy, while 2 KL divergences align student and teacher intermediate features. The total loss is a weighted sum, with a distillation weight of 0.003. Predictions are softened via temperature-scaled softmax before computing KL divergence, smoothing the distribution and reducing sensitivity to noise. This allows the network to learn from both labels and teacher-guided feature alignment. The total loss is shown in formula 11:

$$\text{Loss}=\text{BCELoss}\left(\text{ypred},\text{ytrue}\right)+\lambda \left(\text{KL}\left(\text{student}1,\text{teacher}\right)+\text{KL}\left(\text{student}2,\text{teacher}\right)\right)$$ (11)

where *λ* = 0.003 is the distillation weight, *ypred* and *ytrue* are model predictions and true labels, and student1,2 and teacher denote intermediate features of the student and teacher models.

## 3 Experiment and analysis

### 3.1 Model evaluation

The experimental data in this study are based on three human cell lines: GM12878, K562, and IMR90. The datasets primarily include raw Hi-C data and DNase-seq BigWig files used to generate feature matrices, as well as epigenomic data for biological validation of chromatin loops, such as ChIA-PET, HiChIP, or ChIP-seq. Additionally, orthogonal datasets, including CTCF ChIA-PET and H3K27ac HiChIP, were used to annotate positive and negative samples. All data used in this study are available in the Supplementary material.

#### 3.1.1 Model level configuration assessment

To evaluate the effect of different numbers of feature extraction layers on the performance of CNNKSCEC, we compared the model performance under single-layer, double-layer, three-layer, and four-layer configurations in seed = 51. The evaluation results for each configuration are shown in Table 1. The results show that the three-layer configuration achieved better overall performance than the single-layer and double-layer configurations. Although the four-layer configuration showed comparable or slightly better performance on some metrics, it has a more complex structure and may introduce higher computational cost.

**TABLE 1:** Compare the model evaluation metrics for the iterative use of feature extraction modules—CNNC, SCConv, and ECHybridAddition—across various iterations. (seed = 51).

Metric	Single-layer	Double-layer	Three layers	Four layers
Precision	0.8916	0.8955	0.9059	0.8904
Recall	0.9003	0.9032	0.8976	0.9075
Accuracy	0.9342	0.9364	0.9385	0.9358
F1-score	0.8959	0.8993	0.9019	0.8989
PR AUC	0.9116	0.9146	0.9180	0.9135

To further improve the reliability of the results, we repeated the experiments for each configuration using 10 random seeds. For each random seed, the dataset was split into training and validation sets at an 8:2 ratio, and the same split was used for all configurations under that seed. As shown in Supplementary Table S1, the three-layer configuration achieved the highest mean F1-score of 0.9000 ± 0.0017, with a 95% confidence interval of (0.8988, 0.9012]. In comparison, the F1-score of the single-layer, double-layer, and four-layer configurations were 0.8976 ± 0.0014, 0.8998 ± 0.0015, and 0.8997 ± 0.0016, respectively. Further paired statistical tests showed that the three-layer configuration significantly outperformed the single-layer configuration in terms of F1-score after Holm correction, with p = 0.0383. However, the differences between the three-layer configuration and the double-layer or four-layer configurations were not statistically significant.

For the secondary metric PR-AUC, as shown in Supplementary Table S2, the three-layer configuration also achieved the highest mean value of 0.9155 ± 0.0018, with a 95% confidence interval of (0.9142, 0.9168]. The PR-AUC values of the single-layer, double-layer, and four-layer configurations were 0.9134 ± 0.0012, 0.9155 ± 0.0013, and 0.9152 ± 0.0010, respectively. Although the mean PR-AUC of the three-layer configuration was higher than those of the single-layer and four-layer configurations and comparable to that of the double-layer configuration, the differences among configurations were not statistically significant after Holm correction.

Overall, the three-layer configuration achieved the highest or near-highest average performance in both the primary metric F1-score and the secondary metric PR-AUC, and it significantly outperformed the single-layer configuration in terms of F1-score. Meanwhile, its performance was comparable to those of the double-layer and four-layer configurations, suggesting that further increasing the number of feature extraction layers did not bring clear additional benefits. Therefore, we adopted the three-layer feature extraction structure in this study to achieve a better balance between model representation capability and structural complexity.

#### 3.1.2 Resolution and input channel evaluation

Then, as shown in Table 2, we evaluated the Precision, Recall, Accuracy, F1-score, and PR AUC of the model at 5 kb, 10 kb, and 25 kb resolutions respectively. The results indicate that CNNKSCEC achieves its best overall performance at 5 kb resolution. In addition, higher-resolution data enable the capture of finer-scale chromatin loops and show improved performance in multiple downstream biological analyses, as demonstrated in Section 3.2 and Supplementary Figures S1-S6. Therefore, 5 kb resolution was selected as the primary resolution for subsequent analyses in this study.

**TABLE 2:** Comparison of model evaluation metrics at different resolutions. (seed = 42).

Metric	5 kb	10 kb	25 kb
Precision	0.8894	0.8650	0.8053
Recall	0.9093	0.8775	0.8502
Accuracy	0.9359	0.9268	0.9098
F1-score	0.8993	0.8712	0.8272
PR AUC	0.9136	0.8885	0.8468

In addition, we further performed a statistical significance analysis comparing the CNNKSCEC, model using dual-channel inputs, namely, Hi-C, and DNase-seq data, with the single-channel model using only Hi-C, data. The corresponding results are provided in Supplementary Table S3. The results show that the incorporation of DNase-seq signals significantly improved model performance, further validating the effectiveness of the dual-channel input strategy.

#### 3.1.3 Evaluation of the feature-level self-distillation model

To evaluate the effect of the feature-level self-distillation module on model performance, we compared CNNKSCEC with two ablated settings: teacher-only mode and student-only mode. It should be noted that the teacher in this study is not an independently trained large teacher network, but refers to the integrated feature representation obtained by fusing the two convolutional branch features in the final CNNC module. The student refers to the two convolutional branch features constrained by this integrated feature. Therefore, the ablation experiments were designed according to the feature-level self-distillation structure. The specific setup of the ablation experimental models are as follows:

CNNKSCEC/Full model: The complete CNNKSCEC model adopts a three-stage iterative structure. Each stage consists of the CNNC, SCConv, and ECHybridAddition modules, which are used to progressively perform feature extraction and feature enhancement. In the final stage, the integrated features obtained after branch fusion are used for final classification. Meanwhile, these integrated features are also used as the supervision target in feature-level self-distillation, where a KL-divergence-based distillation loss is employed to constrain the outputs of the two student branches. Therefore, the training loss of the complete model consists of both the BCE classification loss and the KL distillation loss.

Teacher-only mode: In the teacher-only mode, the model keeps the integrated feature branch for training and prediction, but removes the feature-level self-distillation constraints on the two student branches. In this setting, only the final prediction produced by the integrated feature is supervised by the BCE loss, and no KL distillation loss is introduced.

Student-only mode: In the student-only mode, only the outputs of the two student branches are used for training and prediction, and the integrated feature representation is no longer used as the self-distillation target. Specifically, an independent MLP classifier is added after each student branch to map the extracted branch-specific features into prediction logits, which are then converted into prediction probabilities through a sigmoid function. The final prediction is obtained by averaging the prediction probabilities from the two student branches. During training, the outputs of the two student branches are independently supervised by the ground-truth labels, and each branch is optimized using the BCE loss function. Therefore, the total training loss in the student-only mode is defined as the sum of the BCE losses from the two student branches.

First, the three settings were evaluated using the training and validation sets generated by the same random seed. As shown in Table 3, CNNKSCEC achieved the highest F1-score of 0.8993, outperforming teacher-only mode and student-only mode, which obtained F1-score of 0.8987 and 0.8979, respectively. CNNKSCEC also achieved the highest Recall of 0.9093. Although teacher-only mode and student-only mode showed slightly higher Precision and PR-AUC, the overall performance of the three settings was comparable.

**TABLE 3:** Performance evaluation comparison of different models. (seed = 42).

Metric	CNNKSCEC	Teacher-only	Student-only
Precision	0.8894	0.8981	0.9015
Recall	0.9093	0.8993	0.8942
Accuracy	0.9359	0.9362	0.9360
F1-score	0.8993	0.8987	0.8979
PR AUC	0.9136	0.9145	0.9145

To further assess the stability of the results, we repeated the experiments using 10 random seeds. For each random seed, the dataset was split into training and validation sets at an 8:2 ratio, and the same split was used for all model settings under that seed. F1-score was used as the primary evaluation metric for paired statistical testing with Holm correction, as shown in Supplementary Table S4. PR-AUC was used as a secondary metric for supplementary analysis, as shown in Supplementary Table S5. The statistical tests showed that the differences between CNNKSCEC and teacher-only mode, as well as between CNNKSCEC and student-only mode, were not significant in terms of either F1-score or PR-AUC.

Since the overall estimated performance of the models with and without feature-level self-distillation was comparable, we reported the results of both settings. Specifically, the main experimental section presents the results of the complete CNNKSCEC model, which includes the feature-level self-distillation mechanism. To ensure the completeness and transparency of the experimental analysis, we also provided the results of the model without feature-level self-distillation, namely, the teacher-only mode, in the Supplementary material. In this setting, only the integrated feature obtained by fusing the two convolutional branches is used for training and prediction, without applying feature-level constraints from the integrated feature to the two convolutional branches.

The supplementary results show that the model without feature-level self-distillation achieved comparable predictive performance to CNNKSCEC. Therefore, CNNKSCEC was selected as the final model in this study. It should be noted that the feature-level self-distillation module serves mainly as an auxiliary optimization component. The results obtained under the non-distillation setting have been reported in Supplementary Material S9-S18.

### 3.2 Results analysis in GM12878

To evaluate the effectiveness of CNNKSCEC, we compared it with other chromatin loop prediction methods using Hi-C and epigenomic features, including DLoopCaller (Siguo et al., 2022), CGLoop (Wang et al., 2025), Peakachu (Salameh et al., 2020), Chromosight (Cyril et al., 2020), Mustache (Ardakany et al., 2020), YOLOOP (Chen et al., 2024), SIP and GILoop on GM12878, K562, and IMR90 cell lines. The evaluation focused on enrichment of target factors within predicted loop regions, overlap between methods, peak aggregation, and distance distribution. Other tools were run with default parameters. With a clustering threshold of 0.97, CNNKSCEC detected 3,185, 1,431, and 2,232 loops on chromosomes 20–22 in GM12878, K562, and IMR90, respectively. The results indicate that CNNKSCEC effectively identifies chromatin loops supported by multi-omic data.

#### 3.2.1 Enrichment analysis

Chromatin loop formation typically relies on the coordinated action of specific protein factors and epigenetic modifications. To assess the accuracy of loop predictions, enrichment analysis of target factors is commonly used. By examining the enrichment of known loop-associated factors (e.g., CTCF (Phillips and Corces, 2009; Li et al., 2020), H3K27ac, RAD21, SMC1 (Nasmyth and Haering, 2009; Gligoris and Löwe, 2016) at predicted loops, the biological relevance of the predictions can be evaluated. Additionally, promoters and enhancers (Krivega and Dean, 2012), as key gene regulatory elements, influence gene expression and are critical for development and disease processes. Their enrichment reflects the functional relevance of chromatin loops.

**FIGURE 2:**
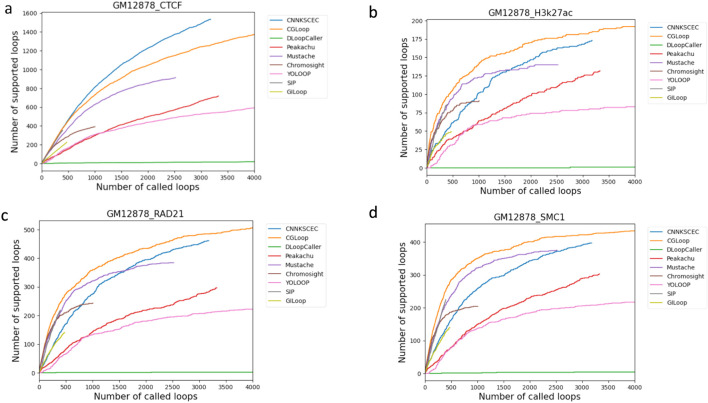
Structural protein enrichment of different tools. Number of chromatin loops supported by CTCF **(a)**, H3K27ac **(b)**, RAD21 **(c)**, and SMC1 **(d)** across different tools.

In this study, we first analyzed the number of predicted loops supported by paired CTCF, H3K27ac, RAD21, and SMC1 across CNNKSCEC and other methods. As shown in Figure 2, compared to other methods, CNNKSCEC-predicted loops exhibit higher enrichment of CTCF and show consistent co-enrichment with multiple loop-associated factors, including H3K27ac, RAD21 and SMC1. These enrichment patterns indicate that CNNKSCEC predictions are preferentially associated with known structural and regulatory components of chromatin loops, rather than representing random chromatin contacts.

**FIGURE 3:**
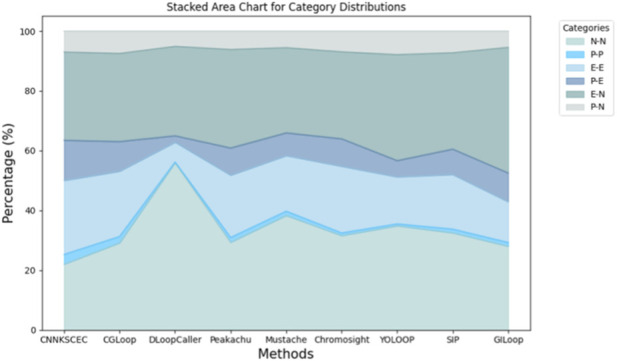
Proportions of regulatory element enrichment across different tools.

To analyze promoter-enhancer interactions within chromatin loops, we first counted the number of promoters or enhancers enriched in the neighborhoods of each predicted loop anchor. Figure 3 shows the enrichment proportions of regulatory elements, where P indicates promoters, E indicates enhancers, and N indicates non-regulatory elements. The results show that, compared with other tools, CNNKSCEC predicts the fewest N-N loops, indicating that its predicted loop anchors are more frequently associated with promoters or enhancers. This suggests that the chromatin loops identified by CNNKSCEC are more likely to be involved in transcriptional regulation, without implying that N-N loops lack biological relevance.

#### 3.2.2 Peak analysis

**FIGURE 4:**
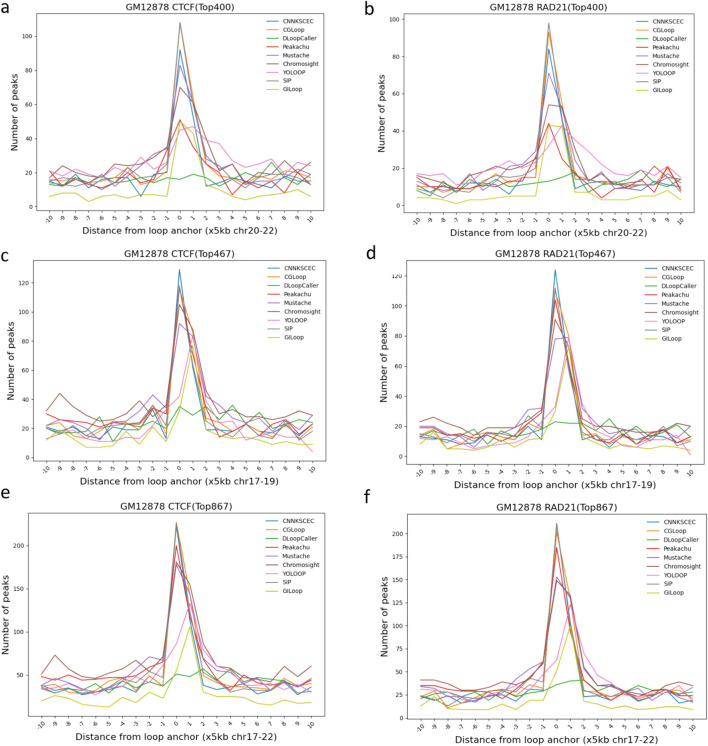
Peaks in the chromatin loop anchor region detected by different tools. **(a, b)** show the CTCF and RAD21 signal peaks, respectively, at the anchors of the top 400 chromatin loops ranked by confidence scores on chromosomes 20–22. **(c, d)** show the CTCF and RAD21 signal peaks, respectively, at the anchors of the top 467 chromatin loops ranked by confidence scores on chromosomes 17–19. **(e, f)** show the CTCF and RAD21 signal peaks, respectively, at the anchors of the top 867 chromatin loops ranked by confidence scores on chromosomes 17–22.

Chromatin loops are three-dimensional genomic structures formed by interactions between two spatially proximal chromatin fragments, and their anchor regions are typically enriched with key protein factors such as CTCF and RAD21. To ensure a fair comparison among different methods, we adopted a normalized analysis strategy, in which the smallest number of loops predicted by any evaluated method was used as the reference, and the same number of loops was retained for each method. Specifically, on chromosomes 20–22, SIP predicted the fewest chromatin loops, with a total of 400 loops. Therefore, for each method, the predicted loops were ranked in descending order according to prediction confidence, and the top 400 loops were selected for analysis. We then performed statistical analysis of CTCF ChIP-seq and RAD21 ChIP-seq signals around single-end chromatin loop anchors and visualized the enrichment peak distributions around loop anchor regions. As shown in Figures 4a,b, although the CTCF and RAD21 enrichment peaks at CNNKSCEC-predicted loop anchors were lower than those of CGLoop and SIP, CNNKSCEC still outperformed most existing methods.

In addition, we observed that SIP and GILoop predicted relatively few chromatin loops, which may limit the statistical effectiveness of the normalized analysis. Therefore, we further expanded the prediction and evaluation scope. Specifically, at 5 kb resolution, we followed the same prediction procedure used for chromosomes 20–22 to predict chromatin loops on chromosomes 17–19. On chromosomes 17–19, the smallest number of predicted loops among the evaluated methods was 467. Thus, the top 467 loops ranked by prediction confidence were selected for each method for protein factor enrichment peak analysis. As shown in Figures 4c,d, CNNKSCEC showed significantly stronger CTCF and RAD21 signals at loop anchor regions than in regions outside the anchors, and its peak signals were markedly higher than those of the other methods.

Furthermore, we merged the chromatin loops predicted on chromosomes 17–22. In the merged dataset, the smallest number of predicted loops among the evaluated methods was 867. Therefore, the top 867 loops ranked by prediction confidence were selected for each method for comprehensive analysis. As shown in Figures 4e,f, under the same loop number and evaluation criteria, CNNKSCEC exhibited enrichment peak intensities comparable to those of SIP and CGLoop and significantly higher than those of the other methods. These results indicate that CNNKSCEC can effectively capture anchor-specific protein factor enrichment patterns.

#### 3.2.3 Overlap analysis and unique loops analysis

Different chromatin loop prediction methods often employ distinct algorithms, feature sets, and parameter settings, which can lead to varying predictions for the same chromatin regions. Overlap analysis can assess the consistency of predictions across multiple tools, providing insight into their reliability and accuracy. To evaluate the effectiveness of our method, we compared the chromatin loops predicted by CNNKSCEC with those predicted by other tools.

**FIGURE 5:**
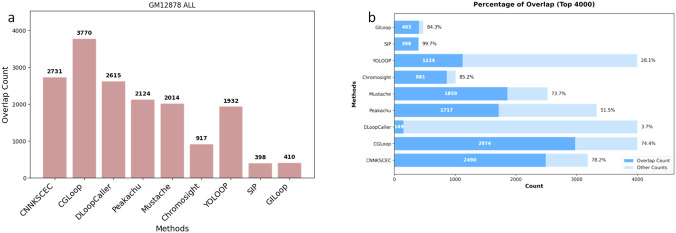
Overlap of chromatin loop predicted by different tools. Figure **(a)** shows the overlapping analysis between all prediction loops and Figure **(b)** shows the overlap percentage of each tool’s confidence scores among the top 4,000 chromatin loops.

We performed overlap visualization of chromatin loops predicted by multiple tools using Chebyshev distance. Figure 5a shows the number of overlapping loops between each tool and others, while Figure 5b the overlap proportions for the top 4,000 high-confidence chromatin loops predicted by each tool, ranked in descending order of their confidence scores (e.g., Mustache loops are ranked by 1-FDR). For methods with fewer than 4,000 predicted loops, the entire output file was utilized for the analysis. The results indicate that CNNKSCEC exhibits a high degree of consistency with other tools.

In addition, we analyzed the support of unique chromatin loops predicted by CNNKSCEC and other methods using CTCF, RAD21, and SMC3 ChIP-seq signals. As shown in Table 4, more than 98% of the loops uniquely identified by CNNKSCEC are supported by these three classical chromatin loop-associated proteins, a proportion that is significantly higher than or comparable to those of other methods. This indicates that the additional loops predicted by CNNKSCEC are not random noise, but are supported by well-established structural proteins. It should be noted that GILoop and SIP detected only a small number of unique loops, with 68 and 2 loops, respectively. Since all of these unique loops were supported by CTCF, RAD21, and SMC3 signals, both methods achieved a support rate of 100%. In comparison, CNNKSCEC identified a much larger number of unique loops supported by these protein factors, thereby providing a broader set of biologically meaningful chromatin interactions for further investigation.

**TABLE 4:** The proportion of unique loops predicted by different tools that are supported by CTCF, RAD21, and SMC3.

Method (unique loops)	CTCF	RAD21	SMC3
CNNKSCEC	98.45%	98.90%	98.90%
CGLoop	98.56%	97.31%	97.79%
YOLOOP	95.48%	94.09%	94.05%
DLoopCaller	89.54%	86.87%	84.93%
Peakachu	96.52%	94.69%	94.03%
Mustachu	87.84%	87.06%	85.69%
Chromosight	97.85%	93.55%	94.62%
GILoop	100.00%	100.00%	100.00%
SIP	100.00%	100.00%	100.00%

Furthermore, we performed a detailed functional categorization analysis of the unique loops. Compared with other methods, as shown in Table 5, CNNKSCEC-specific loops contain a higher proportion of functionally relevant regulatory interactions, including promoter-enhancer (P-E), enhancer-enhancer (E-E), and promoter-promoter (P-P) loops, while exhibiting a relatively lower fraction of loops connecting non-annotated regions (N-N). This suggests that CNNKSCEC is able to recover a subset of chromatin interactions that are missed by other approaches but are more likely to participate in transcriptional regulation.

**TABLE 5:** The proportion of various functional regulatory loops in the unique loops predicted by different tools.

Method (unique loops)	P-E	P-P	E-E	P-N	E-N	N-N
CNNKSCEC	0.1369	0.0243	0.1987	0.0905	0.3024	0.2472
CGLoop	0.0787	0.0144	0.1766	0.0787	0.2994	0.3522
YOLOOP	0.0491	0.0061	0.1499	0.0799	0.3509	0.3641
DLoopCaller	0.0201	0.0019	0.0624	0.0502	0.2965	0.5689
Peakachu	0.0713	0.0191	0.1816	0.0514	0.3358	0.3408
Mustachu	0.0412	0.0078	0.0863	0.0294	0.2176	0.6176
Chromosight	0.0215	0.0108	0.1075	0.0430	0.3226	0.4946
GILoop	0.1176	0.0000	0.1765	0.0735	0.4118	0.2206
SIP	0.0000	0.0000	0.0000	0.0000	0.5000	0.5000

#### 3.2.4 APA analysis

**FIGURE 6:**
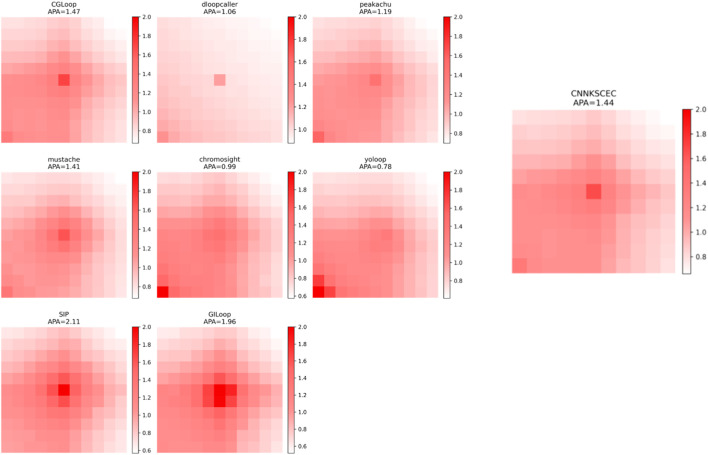
APA scores of chromatin loops predicted by different tools.

Chromatin loops predicted by all tools were analyzed using the apa-analysis method in HiCPeaks to calculate APA scores (Durand et al., 2016). APA plots for loops predicted by each tool are shown in Figure 6. The APA scores of GILoop and SIP were 2.11 and 1.96, respectively, which were higher than those of CGLoop and CNNKSCEC. CGLoop achieved an APA score of 1.47, slightly higher than that of CNNKSCEC, which was 1.44. The higher APA scores of GILoop and SIP may be attributed to the relatively small number of loops predicted by these methods. Their predictions tend to focus on highly conservative loops with strong Hi-C interaction signals, resulting in stronger central enrichment in the APA plots. In contrast, CNNKSCEC predicted a larger number of chromatin loops, including both strong Hi-C signal loops and potential functional loops with relatively weaker Hi-C signals. Therefore, its APA score was slightly lower than those of GILoop, SIP, and CGLoop. Nevertheless, CNNKSCEC still showed clear enrichment at loop centers in the APA plot, indicating that the predicted loops are supported by evident interaction signals. Together with the protein factor enrichment and promoter-enhancer regulatory element analyses, these results suggest that CNNKSCEC can identify a broader set of biologically meaningful chromatin loops rather than only capturing the strongest Hi-C interaction signals.

#### 3.2.5 Distance distribution analysis

**FIGURE 7:**
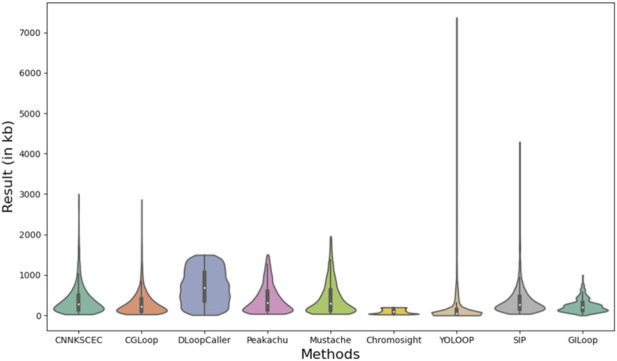
Distance distribution between chromatin loop anchors predicted by different tools.

To examine the distance distribution between the two anchors of predicted chromatin loops, we calculated the distances between paired anchors for loops predicted by different tools. As shown in the Figure 7, most tools identify loop anchors predominantly within 0–250 kb, indicating that the majority of detected loops are short-range. Compared with other tools, CNNKSCEC also identifies some long-range loops. In contrast, loops predicted by DLoopCaller, which also uses multi-feature inputs, are relatively evenly distributed across 0–1,000 kb.

#### 3.2.6 Functional association analysis of promoter-enhancer (P-E) loops

H3K27ac, H3K4me1, and H3K4me3 are classical histone modifications associated with chromatin regulatory states. Specifically, H3K27ac is commonly associated with active enhancers and promoters, H3K4me1 is enriched at enhancers, and H3K4me3 is mainly associated with active promoters. Together, these histone modifications are widely used to characterize regulatory element activity and transcriptional regulatory potential.

**FIGURE 8:**
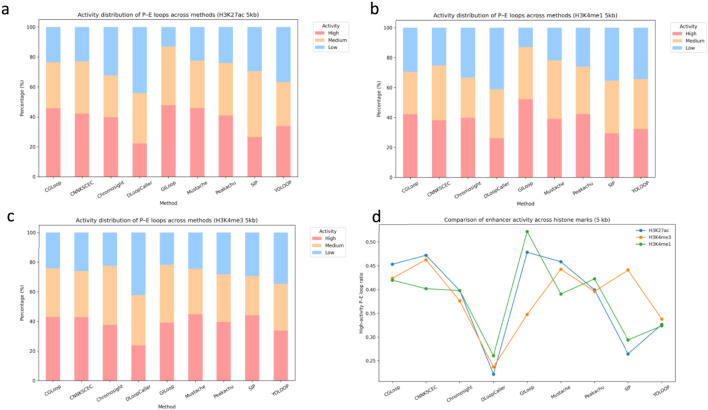
Distribution of promoterenhancer (P–E) loop activity detected by different tools under various histone modifications at 5kb in the GM12878 cell line. **(a)** Distribution of P–E loop activity detected by different tools under H3K27ac. **(b)** Distribution of P–E loop activity detected by different tools under H3K4me1. **(c)** Distribution of P–E loop activity detected by different tools under H3K4me3. **(d)** Comparison of enhancer activity detected by different tools under the three histone modifications.

We identified P-E loops predicted by CNNKSCEC and other methods, and extracted the average ChIP-seq bigWig signal intensity at the enhancer anchors of P-E loops for each histone modification. As shown in Figures 8a–c, CNNKSCEC showed a relatively high proportion of high-activity enhancer anchors and a relatively low proportion of low-activity enhancer anchors across the three histone modifications. Although CNNKSCEC did not achieve the highest value for every individual histone mark, its performance was consistently comparable to or better than most existing methods. These results suggest that CNNKSCEC tends to identify P-E loops associated with functionally active regulatory elements.

Furthermore, we introduced the high-activity P-E loop ratio as a unified quantitative metric to compare different methods across multiple histone modification contexts. As shown in Figure 8d, CNNKSCEC maintained a relatively high and stable high-activity P-E loop ratio across H3K27ac, H3K4me1, and H3K4me3. In contrast, some methods showed larger fluctuations among different histone modifications. Overall, these results indicate that the P-E loops predicted by CNNKSCEC exhibit reliable activity enrichment and relatively strong cross-mark consistency across multiple complementary histone modifications.

To evaluate whether the observed performance gains are primarily attributable to the learned representations rather than the density-based post-processing strategy, we conducted additional ablation analyses (Supplementary Figures S7-S8). The results indicate that although density clustering improves structural consistency, CNNKSCEC without clustering still outperforms existing methods across multiple evaluation metrics. These results demonstrate that the primary source of performance gain lies in the deep feature learning framework of CNNKSCEC rather than in the inherited clustering strategy.

### 3.3 Analysis of chromatin loops across different cell lines

To validate the predictive capability of the CNNKSCEC model across different cell lines, we investigated its biological performance on K562 and IMR90 cell lines. By integrating experimental results from multiple cell lines, we systematically analyzed the cross-cell line prediction ability of the CNNKSCEC model for chromatin loops.

#### 3.3.1 Enrichment analysis and peak analysis

**FIGURE 9:**
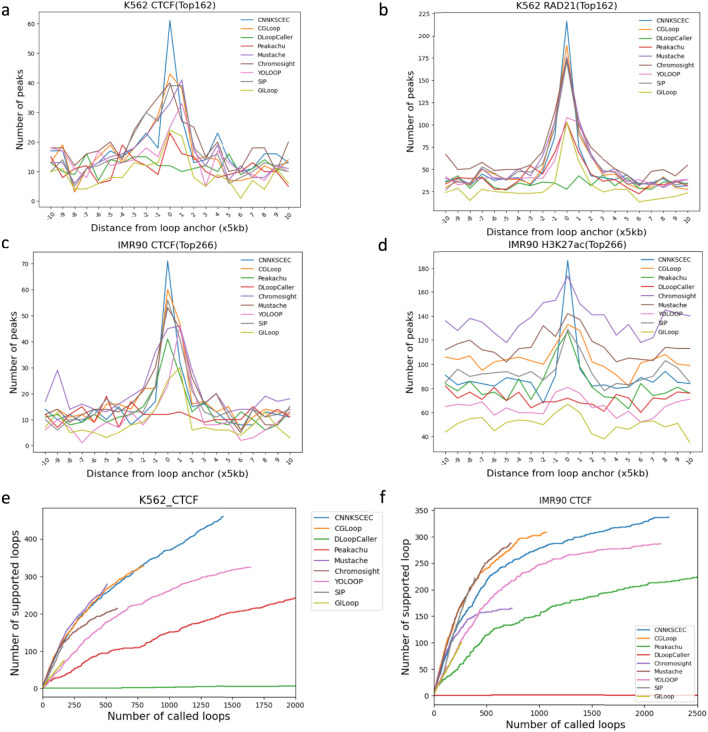
Structural protein enrichment and peak distributions at loop anchor neighborhoods for chromatin loops detected by different tools in K562 and IMR90 cell lines on chromosomes 2022. **(a,b)** Distribution of CTCF and RAD21 at loop anchor neighborhoods detected by different tools in K562 cell line. **(c,d)** Distribution of CTCF and H3K27ac at loop anchor neighborhoods detected by different tools in IMR90 cell line. **(e,f)** Number of CTCF-supported chromatin loops detected by different tools in K562 and IMR90 cell lines.

Similar to the analysis performed in the GM12878 cell line, to ensure a fair comparison among different tools, we used the minimum number of chromatin loops detected by all tools within the same cell line and at the same resolution as a unified standard in both K562 and IMR90 cells. We first analyzed the predicted chromatin loops on chromosomes 20–22. Specifically, in the K562 cell line, SIP detected the fewest chromatin loops, with 162 loops; in the IMR90 cell line, GILoop detected the fewest chromatin loops, with 266 loops. Therefore, based on these fixed numbers of chromatin loops, we compared the peak distributions of relevant protein factors in the neighborhoods of loop anchor regions predicted by different methods. The results are shown in Figures 9a–d.

However, because SIP and GILoop also predicted relatively few chromatin loops on chromosomes 20–22 in the K562 and IMR90 cell lines, we further expanded the scope of prediction and evaluation. Specifically, we analyzed the chromatin loops predicted by different tools on chromosomes 17–19 in the K562 and IMR90 cell lines, respectively, and again used the minimum number of loops predicted by the evaluated methods as a unified standard to assess protein-factor enrichment signals around loop anchors. We then merged the predictions obtained from chromosomes 17–22 and performed a combined analysis following the same procedure described in Section 3.2.2. The results are presented in Supplementary Figure S19. Both extended analyses consistently showed that CNNKSCEC exhibited significantly stronger protein-factor signals at loop anchor regions than in flanking regions, with enrichment peaks markedly higher than those of the other methods.

Furthermore, we compared the number of CTCF-supported chromatin loops detected by CNNKSCEC and other tools in the K562 and IMR90 cell lines, as shown in Figures 9e,f. The results indicate that, in both cell lines, a considerable number of chromatin loops predicted by CNNKSCEC were supported by CTCF signals, further demonstrating that CNNKSCEC can reliably identify chromatin loop structures across different cell lines.

In summary, in both K562 and IMR90 cell lines, the chromatin loop anchor regions predicted by CNNKSCEC showed the highest protein factor enrichment peaks, and a substantial number of predicted loops were supported by CTCF. These findings indicate that CNNKSCEC can stably and reliably identify biologically supported chromatin loop structures across different cell lines, further validating the robustness and generalizability of the proposed method.

#### 3.3.2 Distance distribution analysis

**FIGURE 10:**
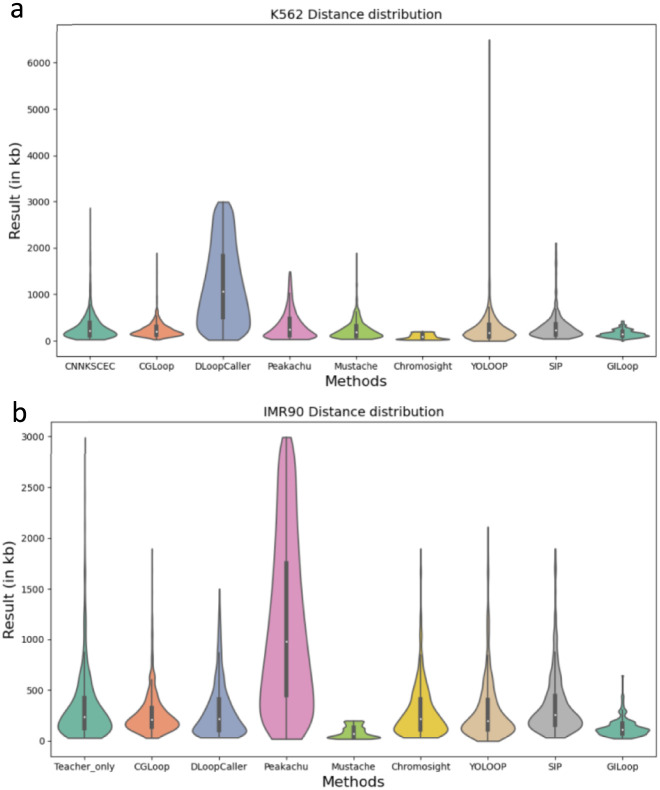
The distribution of distance for different tools in K562 **(a)** and IMR90 **(b)**.

To analyze the distribution of distances between the two anchor points of predicted chromatin loops across different cell lines, we calculated the distances between paired anchor points in loop structures predicted by various tools in the K562 and IMR90 cell lines. As shown in Figure 10, the majority of anchor points identified by these tools were located within the 0–250 kb range, indicating that most detected loops are short-range structures. Additionally, CNNKSCEC not only detected short-range loops but also identified some medium-to long-range loops, a finding consistent with its observations in GM12878.

#### 3.3.3 APA analysis

**FIGURE 11:**
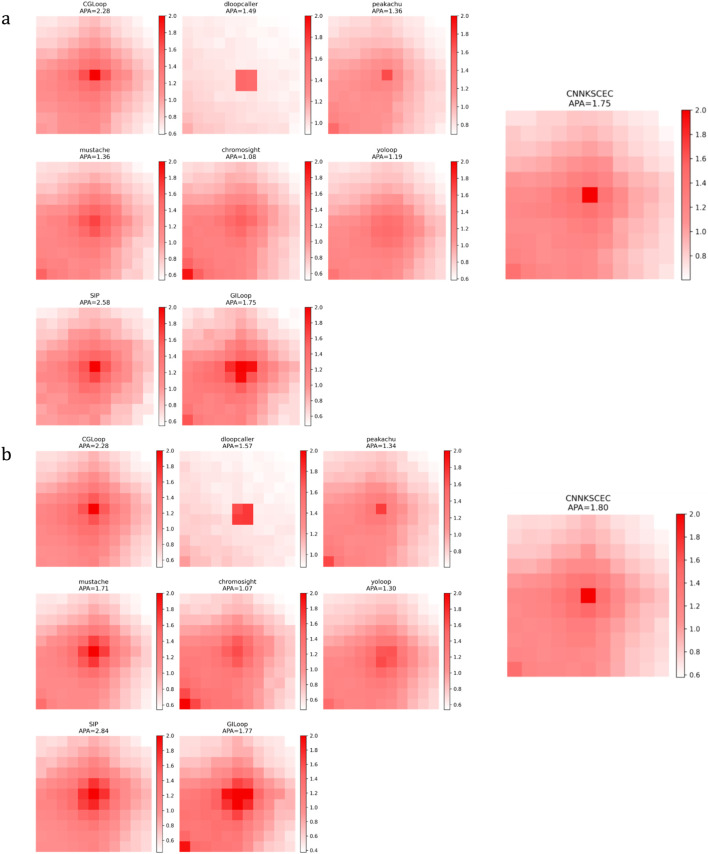
The APA scores of the chromatin loops predicted by different tools in the K562 **(a)** and IMR90 **(b)** cell lines.

In both the K562 and IMR90 cell lines, the APA_score for the chromatin loops predicted by all tools was calculated using the apa-analysis method from HiCPeaks. The APA plots for each tool’s predicted chromatin loops are shown in Figure 11. Consistent with the results observed in the GM12878 cell line, the interaction intensity at the anchor regions of the chromatin loops predicted by CNNKSCEC, although lower than that of CGLoop and SIP, was still higher than that of the other tools. Furthermore, as seen in the figure, CNNKSCEC demonstrated stronger interaction intensity and revealed an interaction effect in the lower-left background.

## 4 Discussion

Deep learning has become a core tool for addressing complex scientific problems and driving technological innovation, thanks to its advantages in automatic feature extraction, high-dimensional data processing, nonlinear modeling, multimodal integration, and transfer learning. Increasingly, researchers are applying deep learning frameworks to the study of the three-dimensional (3D) genome, continuously advancing the field of 3D genomics.

The CNNKSCEC method proposed in this study demonstrates relative advantages in multiple aspects. First, across different evaluation metrics and multiple cell types, CNNKSCEC is able to stably predict chromatin loops with high levels of biological support. In particular, in promoter-enhancer (P-E) loop analysis, the loops predicted by CNNKSCEC show a stronger tendency to connect regulatory elements with well-defined transcriptional regulatory features, and exhibit consistent enrichment of active histone modification signals, including H3K27ac, H3K4me1, and H3K4me3. These results indicate that CNNKSCEC is capable of identifying not only potential spatial chromatin contacts but also regulatory interactions with putative functional significance.

In addition, structural protein support analysis shows that CNNKSCEC-specific loops are highly enriched for classical chromatin loop-associated proteins such as CTCF, RAD21, and SMC3, further suggesting that these predictions are not random noise but represent chromatin interactions with a clear structural basis. Taken together, these findings support the reliability of CNNKSCEC in chromatin loop prediction from both functional and structural perspectives.

Although CNNKSCEC achieves well performance in the current study, there remains room for further improvement. Future work may focus on the following directions: i) incorporating additional epigenetic or transcriptomic data to further enhance the identification of functional chromatin loops; ii) exploring more effective knowledge distillation architectures and training strategies, as the current feature-level self-distillation module showed only modest improvement; iii) simplifying the model architecture while maintaining predictive performance to improve computational efficiency and scalability; iv) exploring more model-adaptive end-to-end loop localization strategies to further reduce reliance on manually defined post-processing rules; and (v) investigating conceptual advances in chromatin loop prediction.

## 5 Conclusion

In this study, we developed a more robust and extensible deep learning-based framework for feature representation and integration, termed CNNKSCEC, building upon existing chromatin loop detection methods. The primary contribution of this study lies in constructing a dual-channel feature input matrix that integrates Hi-C and DNase-seq data, as well as establishing a multi-level feature extraction framework comprising CNNC, SCConv, and ECHybridAddition. To evaluate the effectiveness of the proposed approach, we conducted a series of biological validation analyses, including protein factor enrichment analysis, peak analysis, loop overlap analysis, and aggregate peak analysis (APA). In addition, CNNKSCEC was applied to multiple cell types, including GM12878, K562, and IMR90. The experimental results demonstrate that CNNKSCEC exhibits strong predictive performance, robustness, and cross-cell-type consistency. Notably, CNNKSCEC shows a clear advantage in identifying promoter-enhancer (P-E) regulatory loops with well-defined functional characteristics, making it particularly suitable for functional 3D genome studies and the investigation of regulatory mechanisms. Overall, CNNKSCEC provides a promising tool for high-precision chromatin loop prediction and the biological functional annotation of chromatin interactions.

## Data Availability

The datasets analyzed in this study are publicly available in the GEO and ENCODE repositories and in previously published studies. The Hi-C data for GM12878, K562, and IMR90 are deposited in the GEO repository under accession number GSE63525. The ENCODE datasets used in this study, including DNase-seq, ChIP-seq, and ChIA-PET data for GM12878, K562, and IMR90, are deposited in the ENCODE repository under accession/file IDs ENCFF264NMW, ENCSR000DZN, ENCSR000DZP, ENCFF180LKW, ENCFF682WPF, ENCFF674QZB, ENCFF352SET, ENCFF001THV, ENCFF002ENT, ENCFF001XSU, ENCFF291DOH, ENCFF464KWY, ENCSR000EFJ, and ENCSR002YRE. For the GM12878 cell line, the CTCF ChIA-PET, RAD21 ChIA-PET, H3K27ac HiChIP, and SMC1 HiChIP datasets were obtained from previously published studies by Tang et al. (2015), Heidari et al. (2014), Mumbach et al. (2017), and Mumbach et al. (2016), respectively. Detailed data and information for each cell line are provided in the “Availability of data and material” section of the Supplementary Material.
