# Supplementary material for: CNNKSCEC: a deep learning-based framework for chromatin loop prediction with multi-source feature integration
Source: Front Genet. 2026 Jul 3;17:1850219. doi: 10.3389/fgene.2026.1850219 (PMC13375185; doi:10.3389/fgene.2026.1850219)
Supplement: Supplementary file 1 [file Supplementaryfile1.docx]

a

b

c

d

e

f

g

h


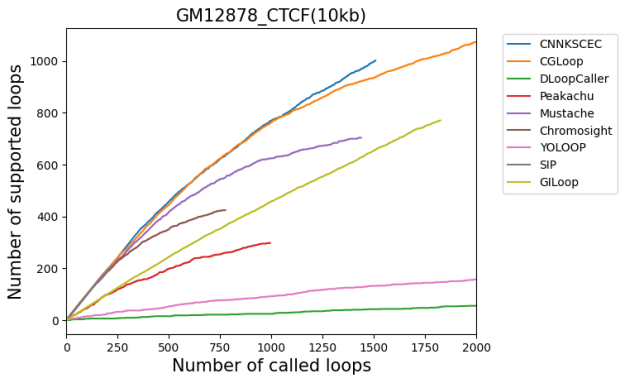

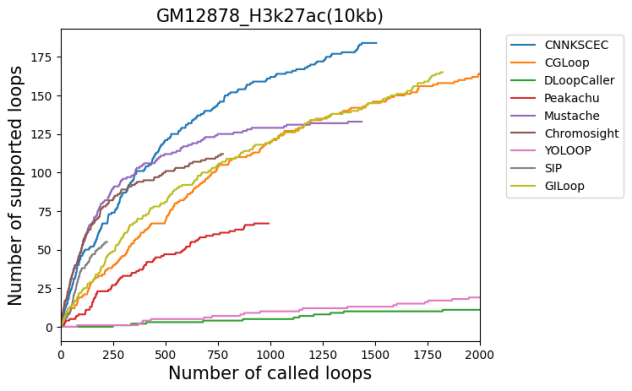

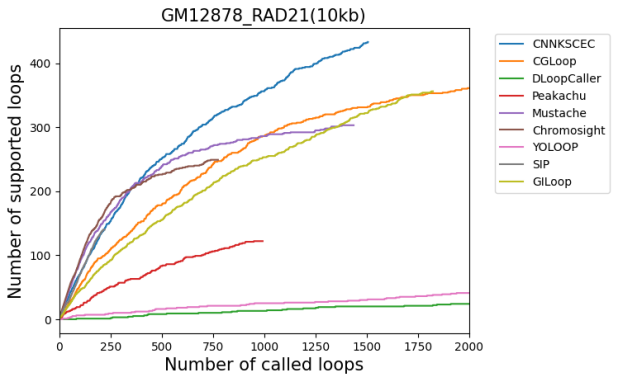

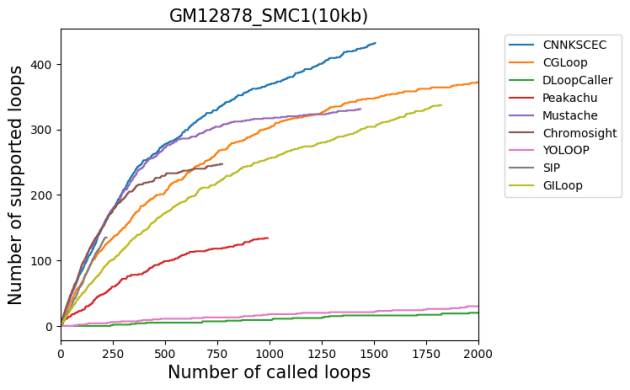

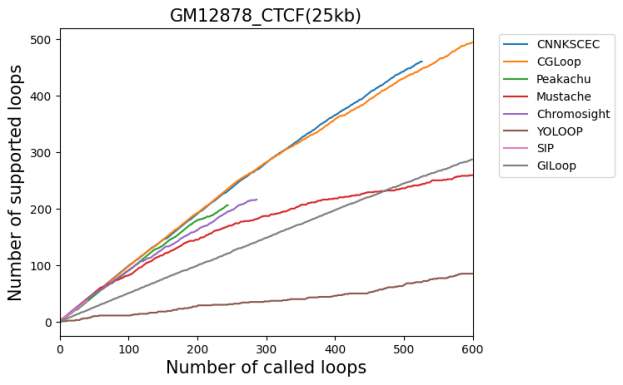

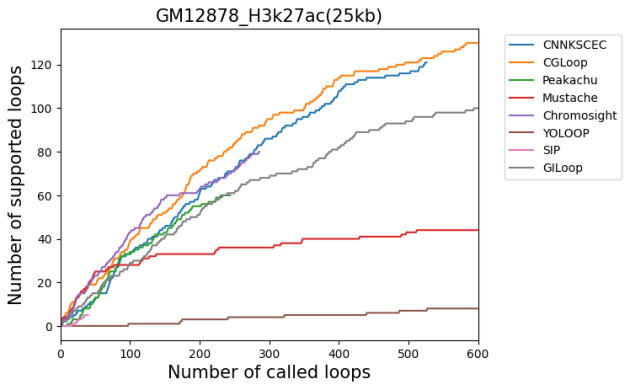

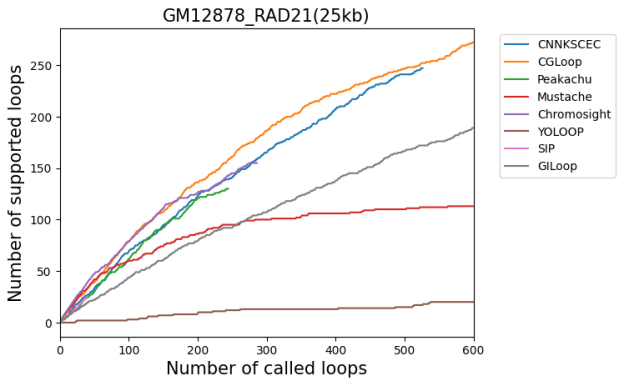

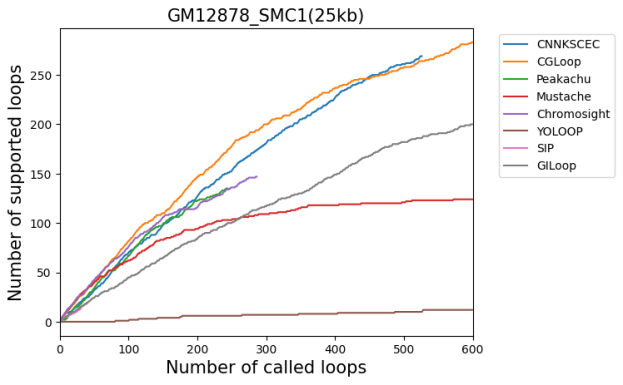


Figure S1. Structural protein enrichment analysis of different tools at 10kb and 25kb.

a

b

c

d


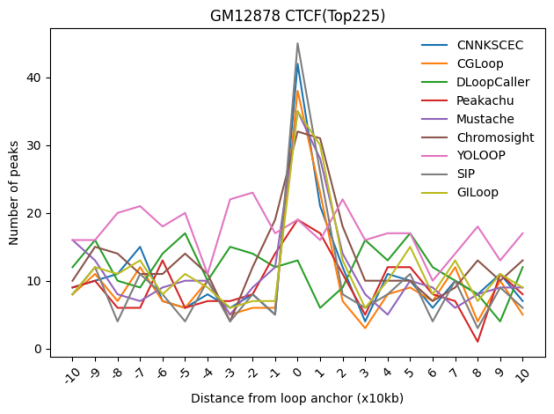

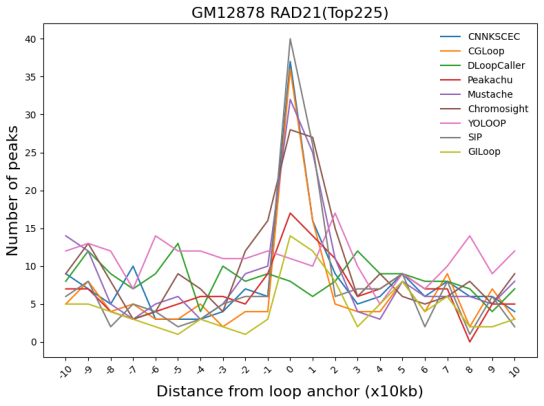

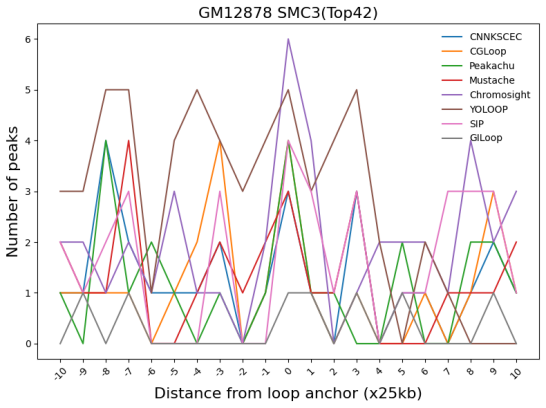

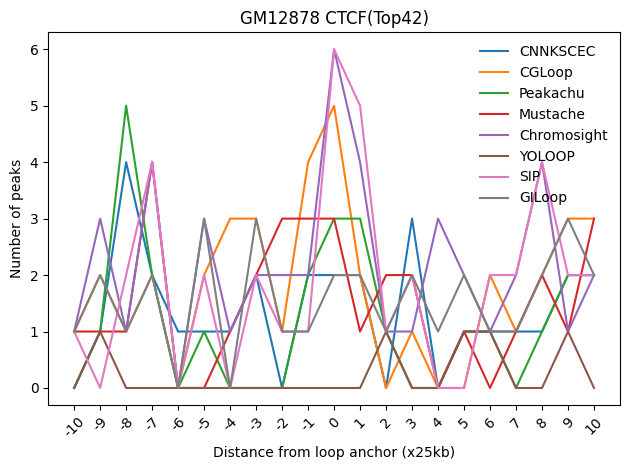


Figure S2. Analysis of CTCF and RAD21 Peaks at chromatin loop anchors Detected by Different Tools at 10kb and 25kb.

a

b

c

d


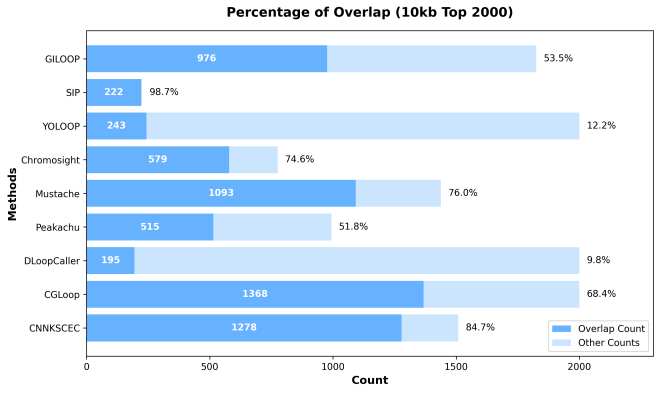

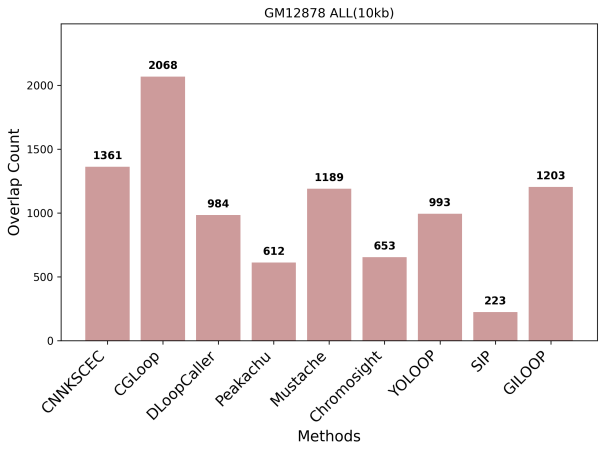

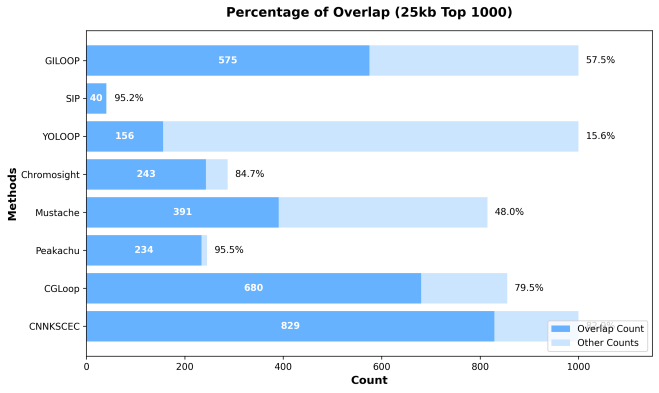

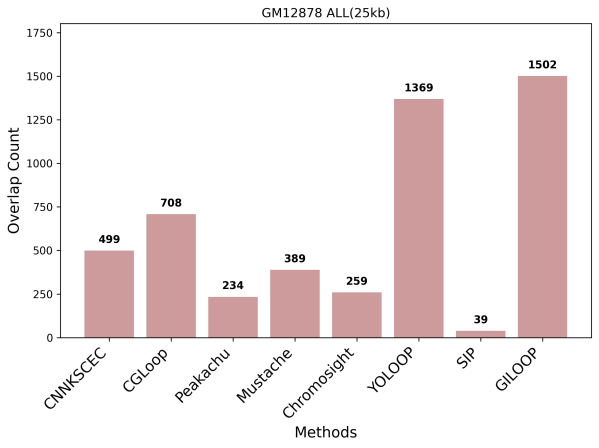


Figure S3. Chromatin loop overlap predicted by different tools at 10kb and 25kb. Figures (a) and (c) show the number of overlaps, while Figures (b) and (d) demonstrate the overlap percentages of the top 2000 (10kb) and top 1000 (25kb) chromatin loops predicted by each tool, ranked by confidence.

a

b


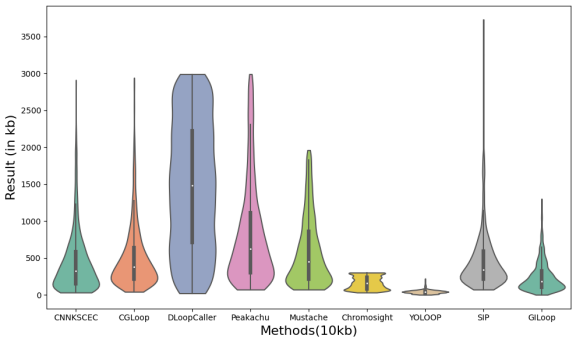

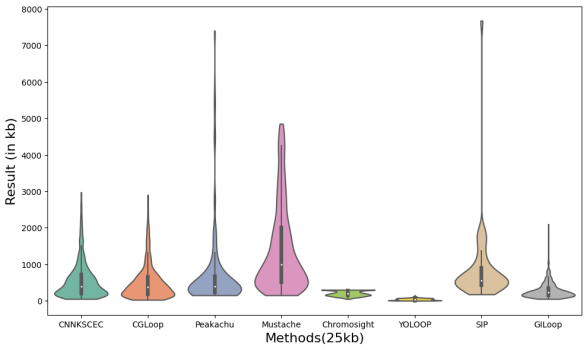


Figure S4. Distribution of chromatin loop anchor distance predicted by different tools at 10kb and 25kb.

a

b


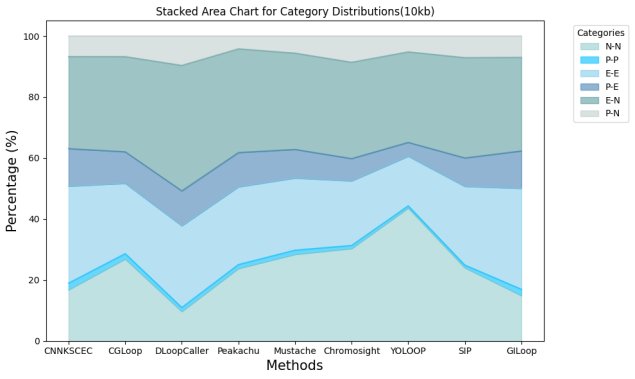

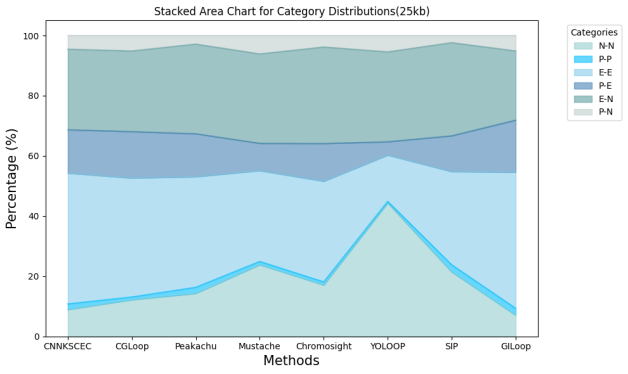


Figure S5. Comparison of regulatory element enrichment ratios among different tools at 10kb and 25kb scales.


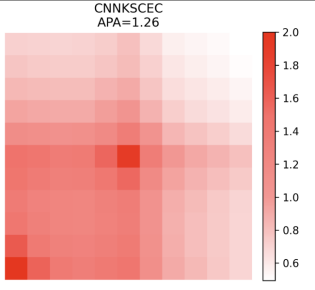

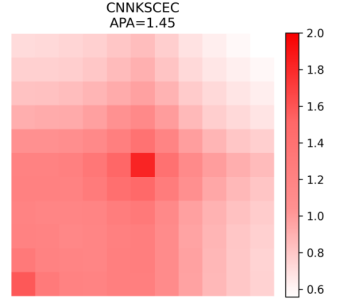

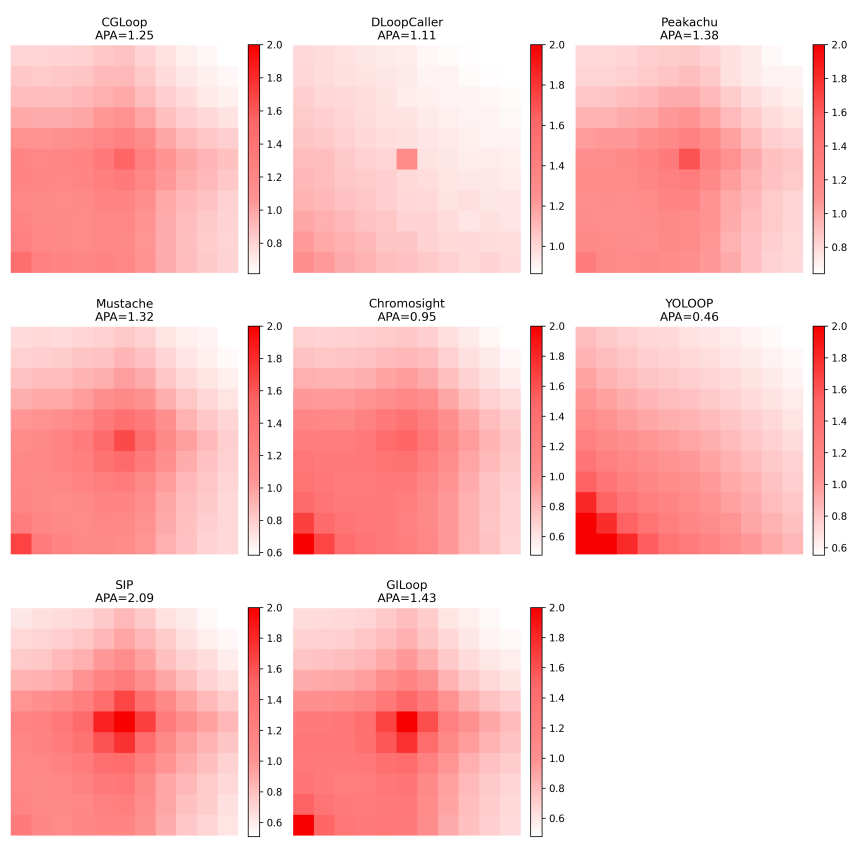

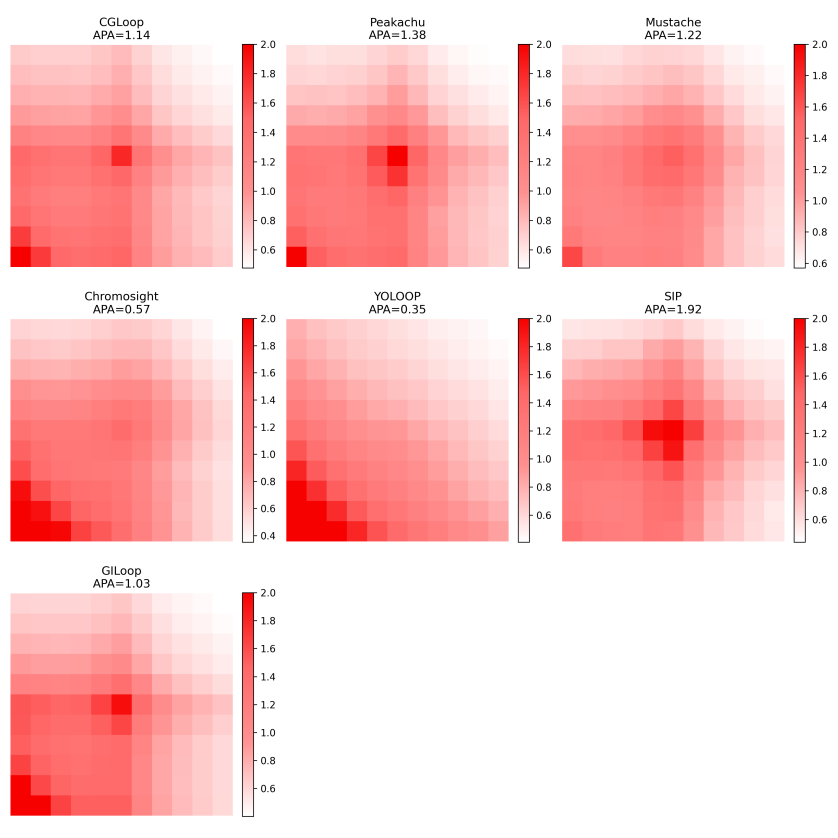


b

a

Figure S6. APA scores for chromatin loops predicted by different tools at 10 kb (a) and 25 kb (b).

a

b

c

d

e

f

g

h


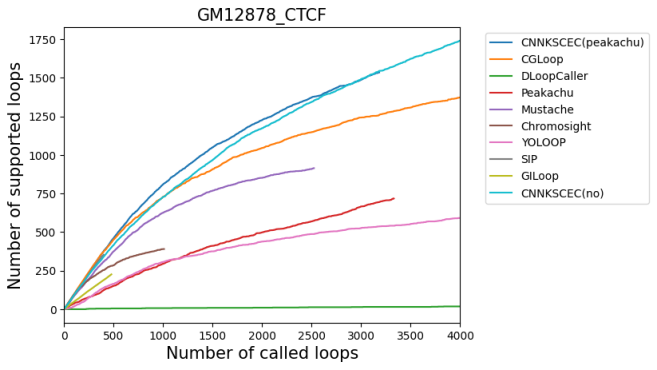

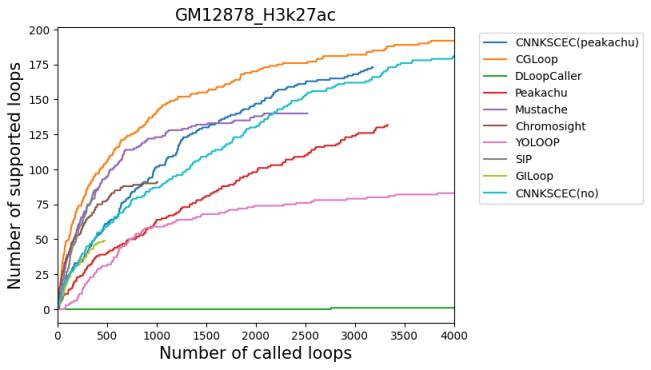

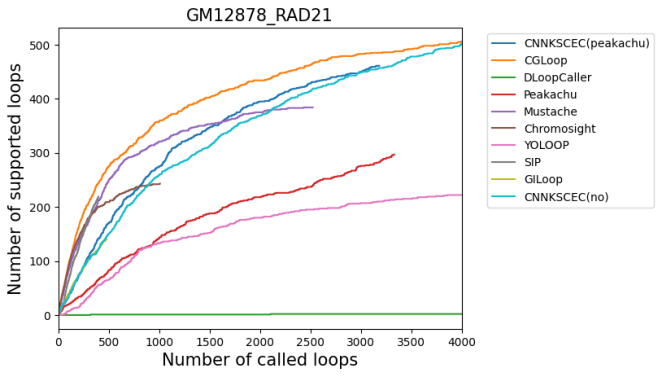

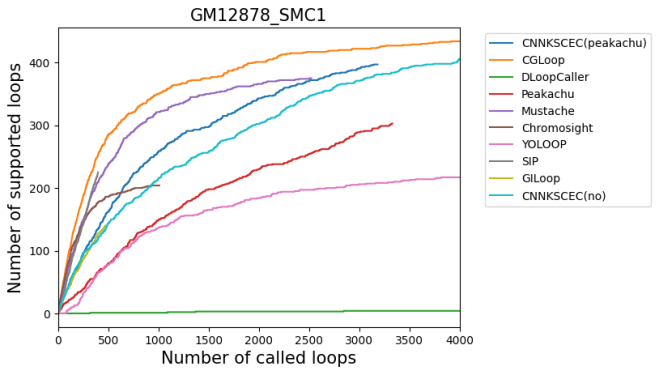

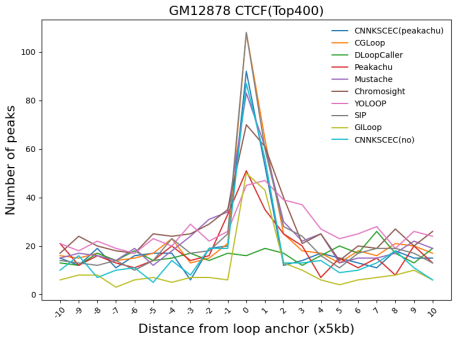

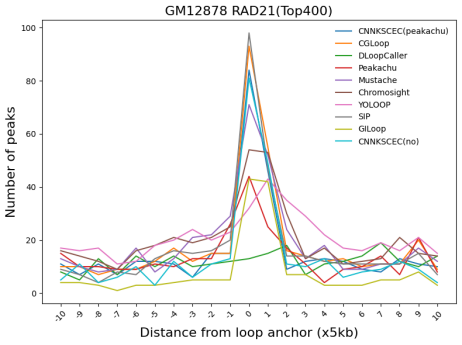

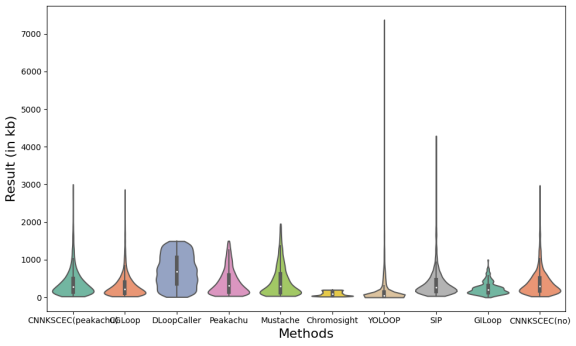

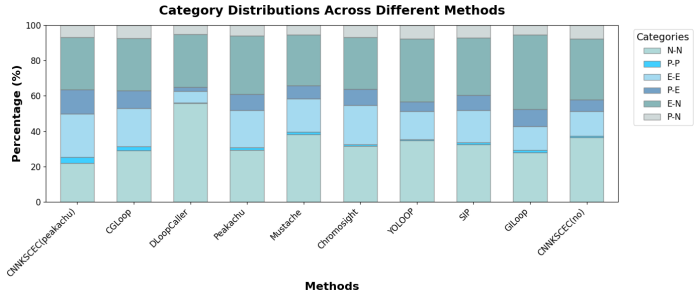


Figure S7. Comparison results of the original prediction results of CNNKSCEC (non-density-dependent clustering), the prediction results of CNNKSCEC (density-dependent clustering), and other existing methods. (a)-(e) Structural protein enrichment analysis; (e)-(f) Peak analysis of protein factors at chromatin loop anchors; (g) Regulatory element enrichment analysis; (h) Chromatin loop distance distribution analysis.


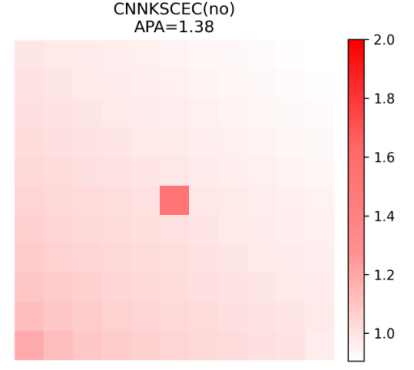

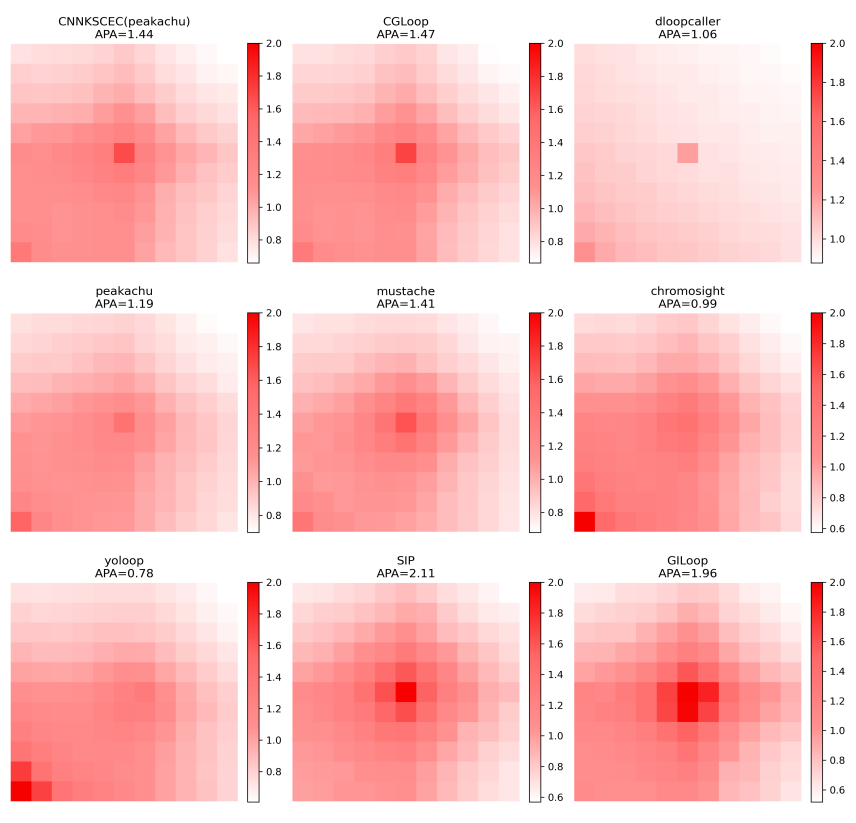


Figure S8. The APA scores of chromatin loops detected by CNNKSCEC (dependency density clustering) and CNNKSCEC (non-dependency density clustering), as well as other tools.

a

b

c

d


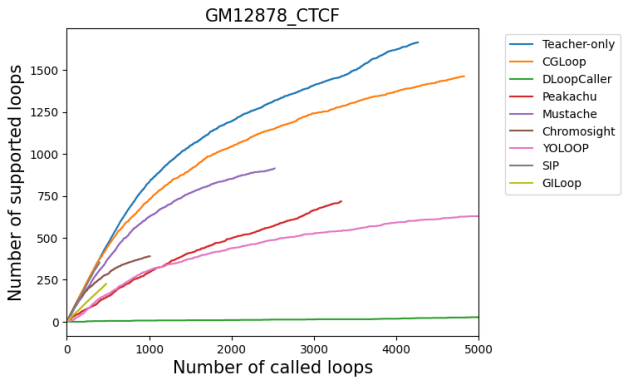

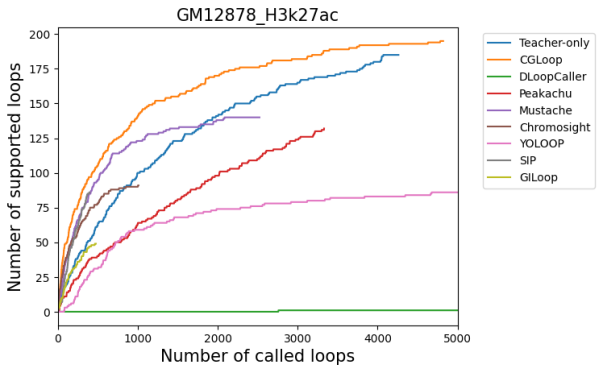

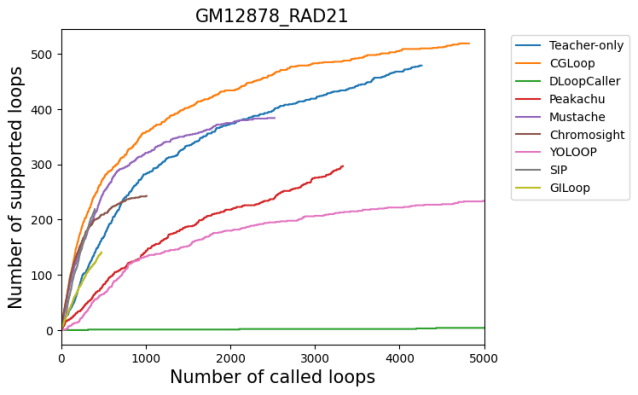

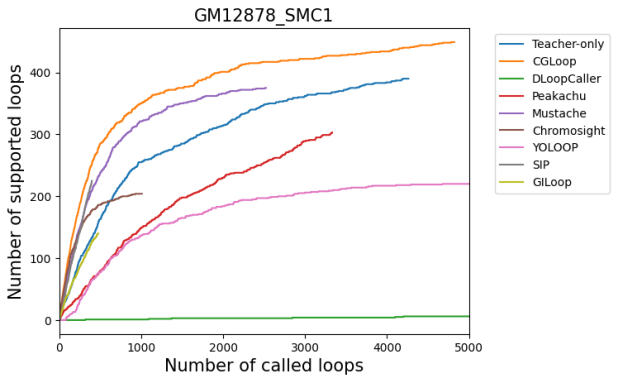


Figure S9. Comparison of protein factor enrichment in chromatin loops detected by the teacher model and other tools


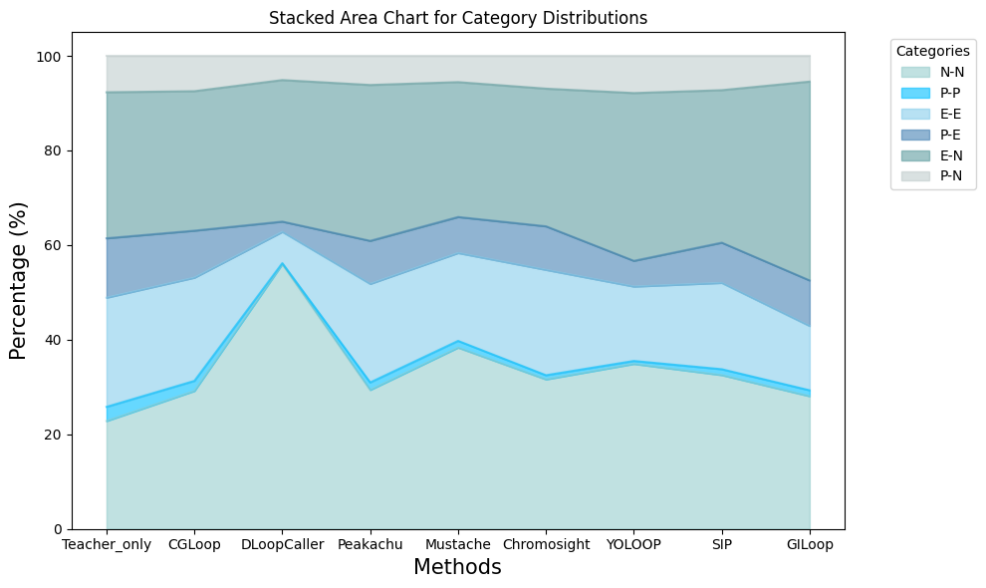


Figure S10. Proportions of regulatory element enrichment in chromatin loops detected by the teacher model and other tools

a

b


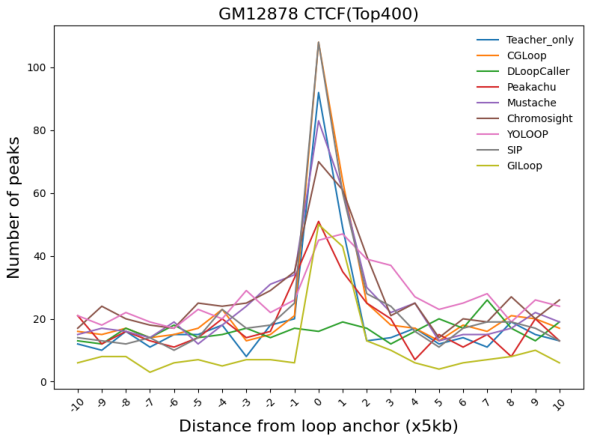

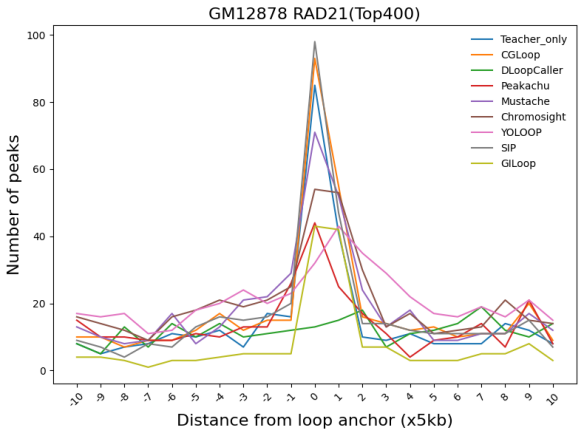


Figure S11. Comparison of protein factor peaks predicted by the teacher model and other tools for chromatin loops

a

b


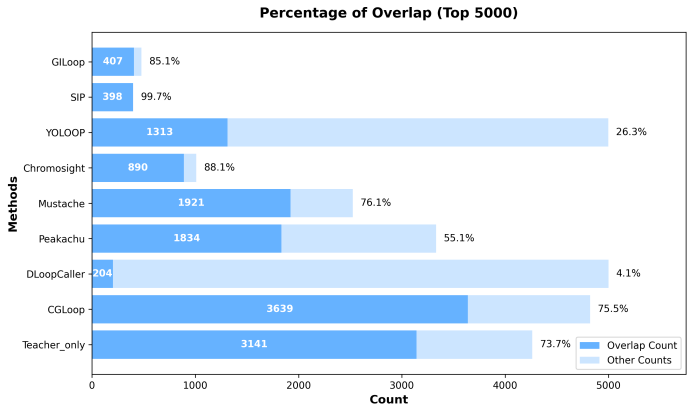

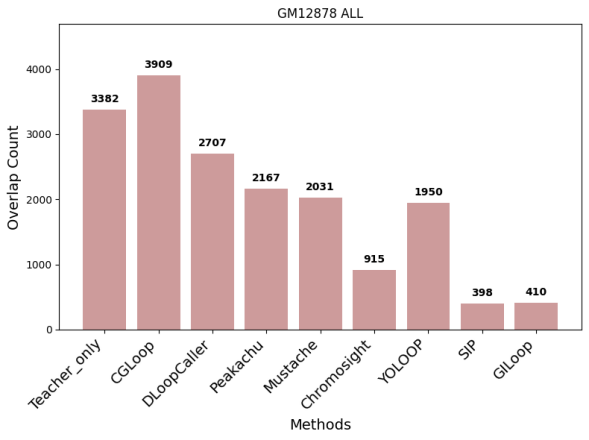


Figure S12. Overlap of chromatin loops predicted by the teacher model and other tools. Figure (a) shows the number of overlaps and Figure (b) shows the overlap percentage of each tool's confidence scores among the top 5000 chromatin loops


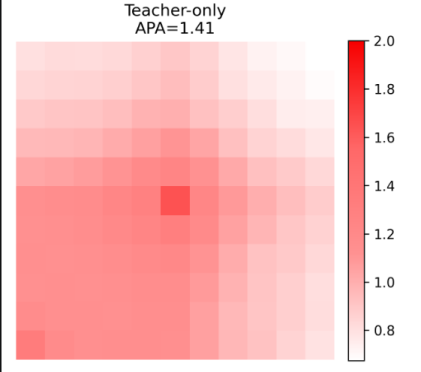

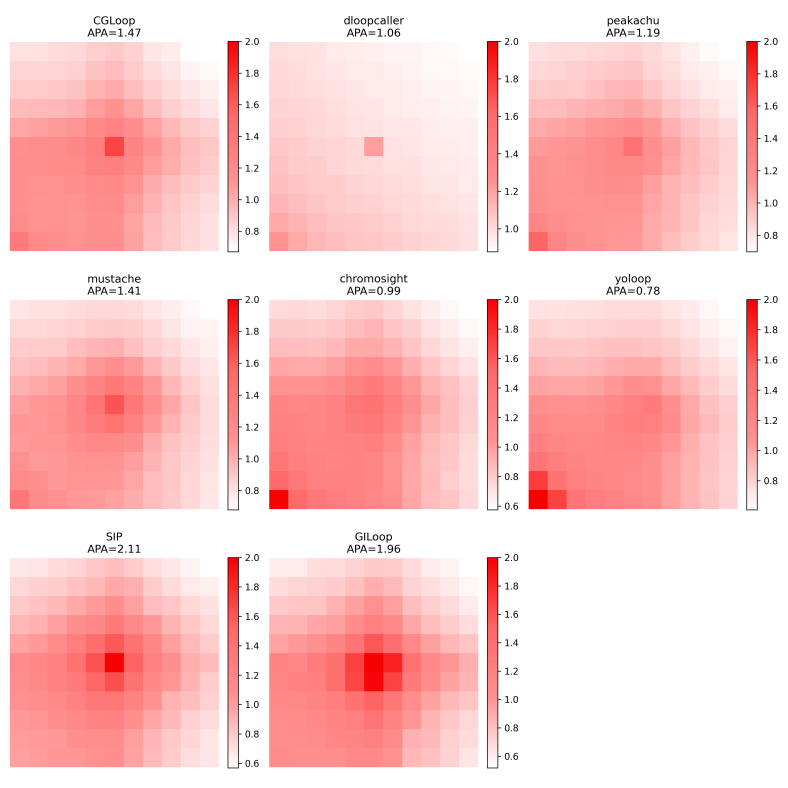


Figure S13. APA scores of chromatin loops predicted by the teacher model and other tools


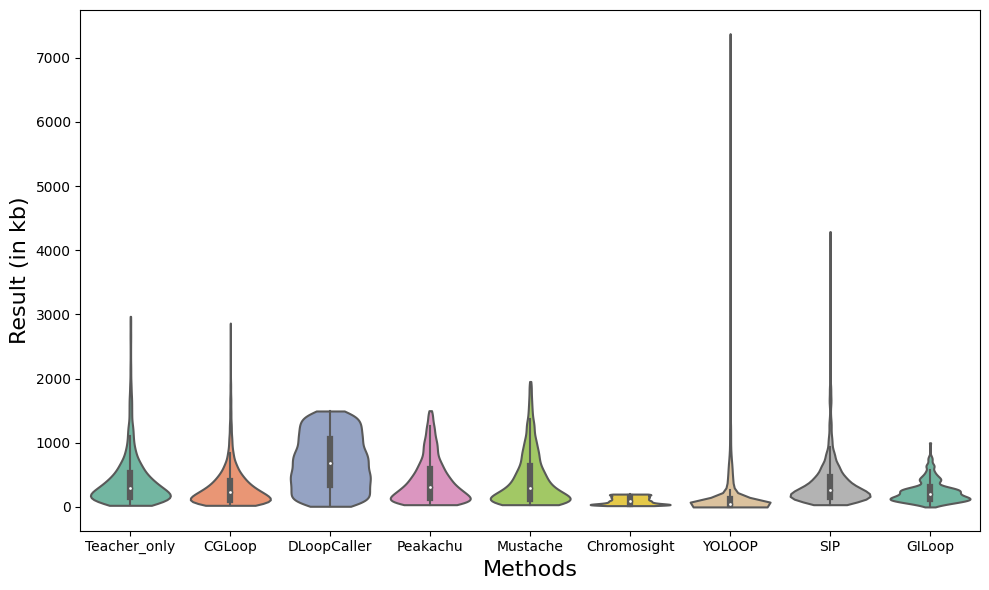


Figure S14. Distance distribution between chromatin loop anchors predicted by the teacher model and other tools

a

bb

c

d

e

f


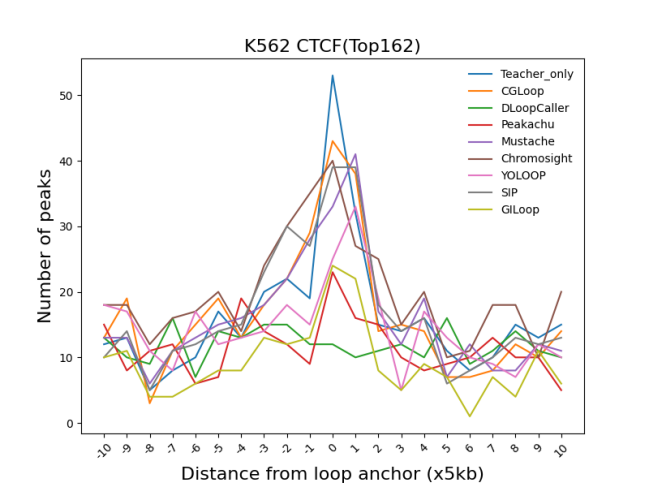

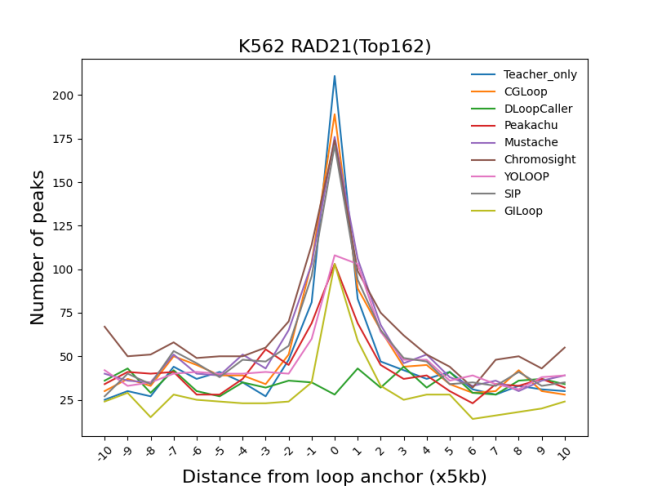

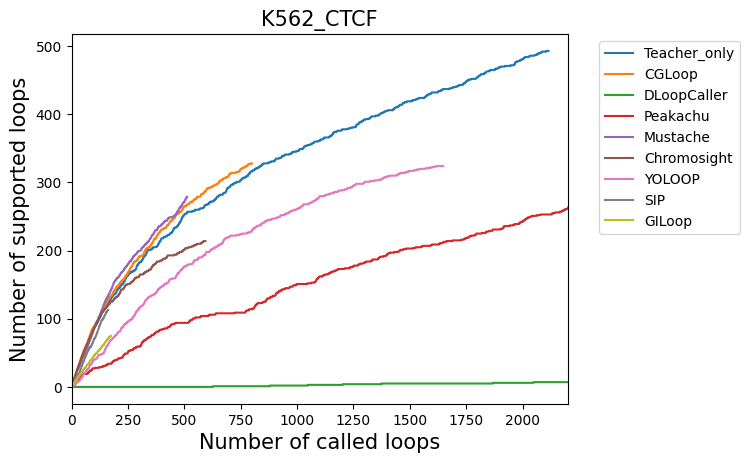

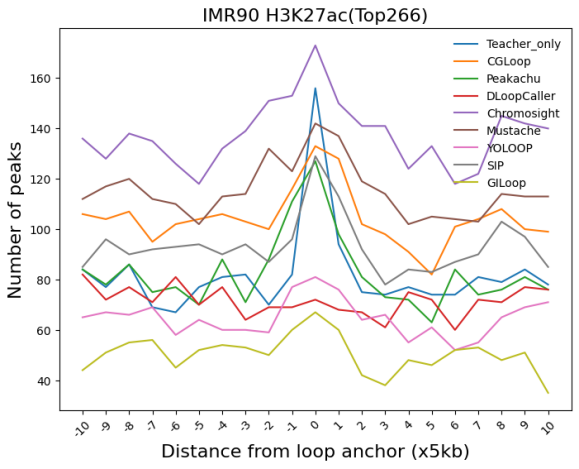

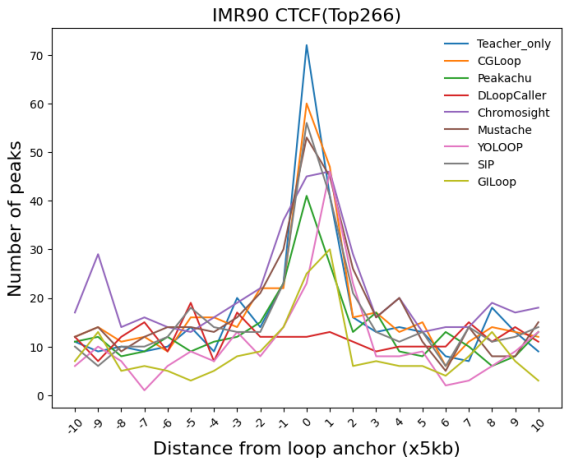

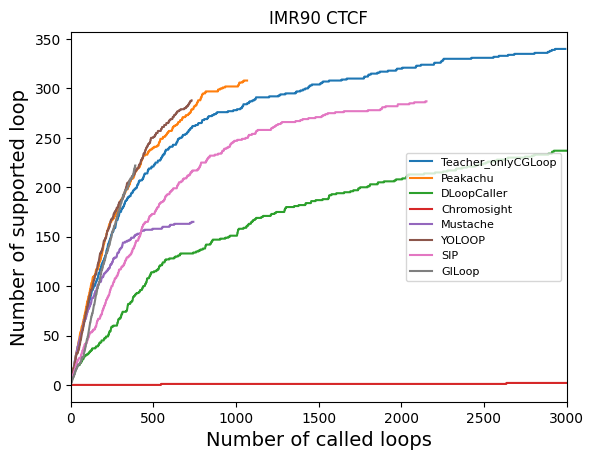


Figure S15. Structural protein enrichment and peak distributions at loop anchor neighborhoods for chromatin loops detected by the teacher model and other tools in other cell lines. a, b, Distribution of CTCF and RAD21 at loop anchor neighborhoods detected by different tools in K562 cells. c, d, Distribution of CTCF and H3K27ac at loop anchor neighborhoods detected by different tools in IMR90 cells. e, f, Number of CTCF-supported chromatin loops detected by different tools in K562 and IMR90 cells

a

b


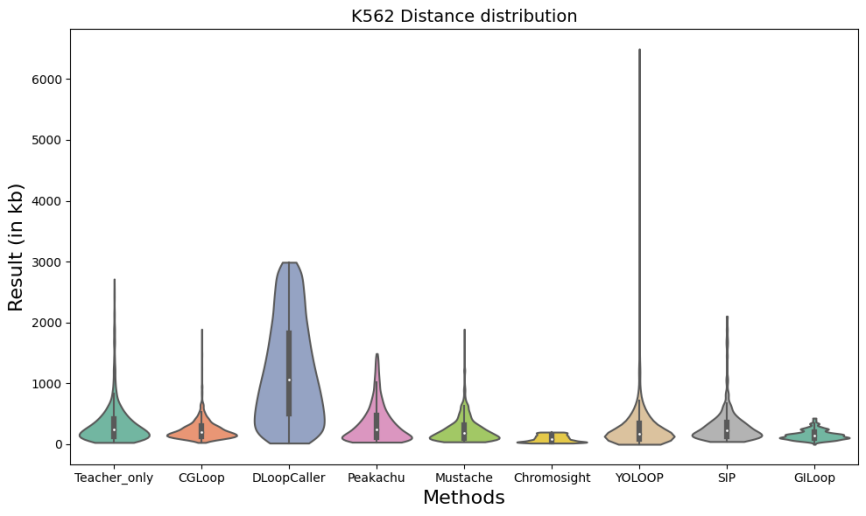

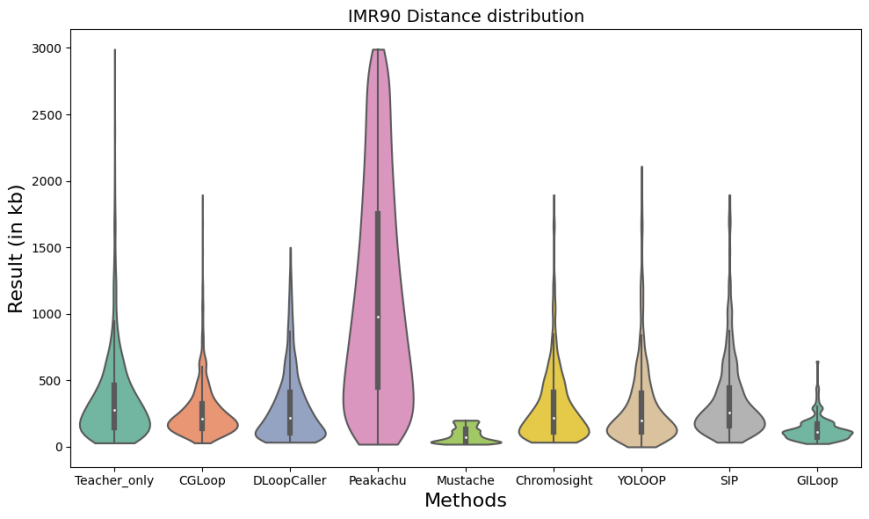


Figure S16. The distribution of distance for the teacher model and other different tools in K562(a) and IMR90(b)


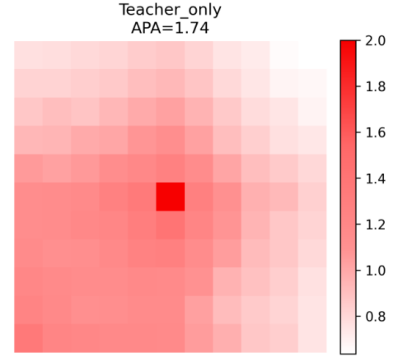

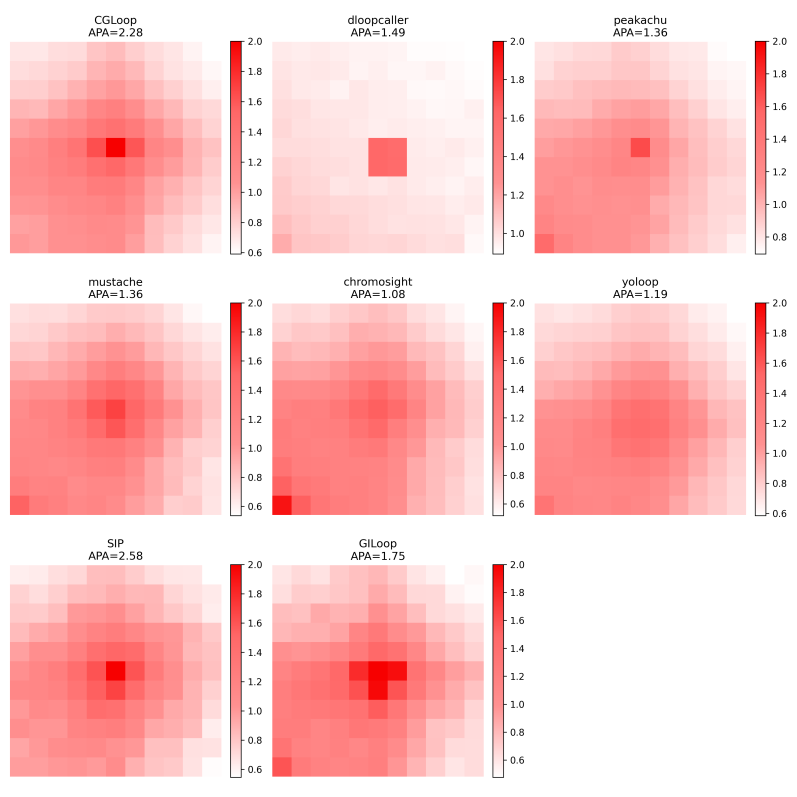


Figure S17. The APA scores of the chromatin loops predicted by the teacher model and other tools in the K562 cell line


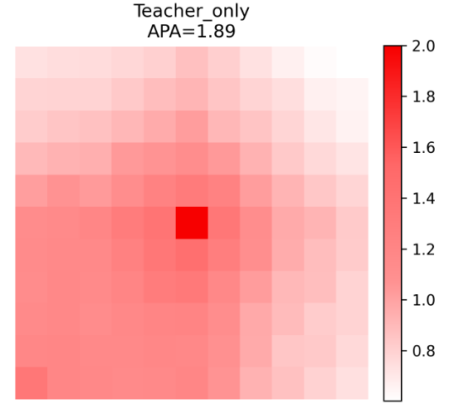

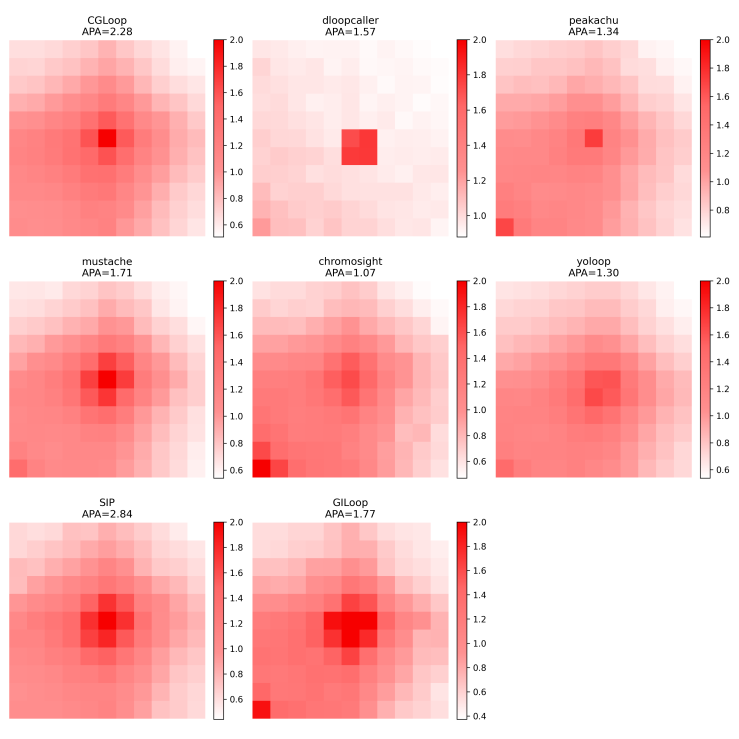


Figure S18. The APA scores of the chromatin loops predicted by the teacher model and other tools in the IMR90 cell line

a

b

c

d

e

f

g

h


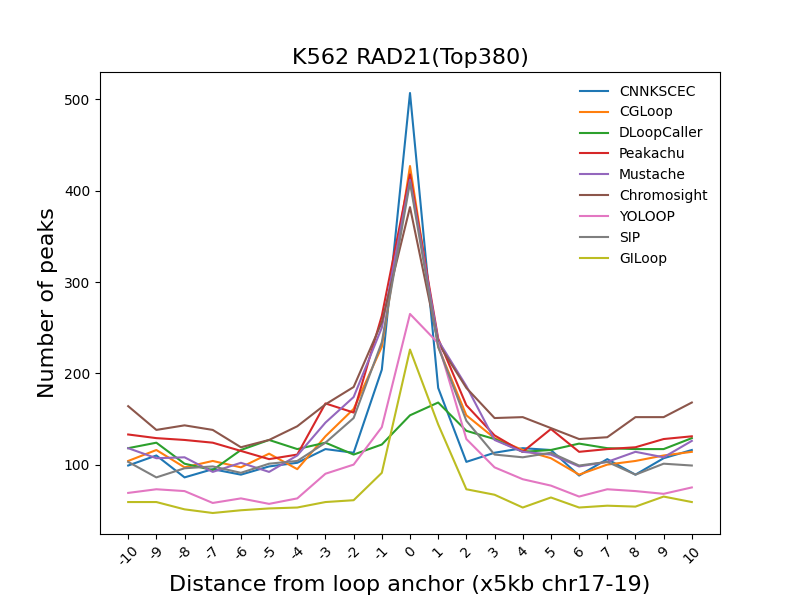

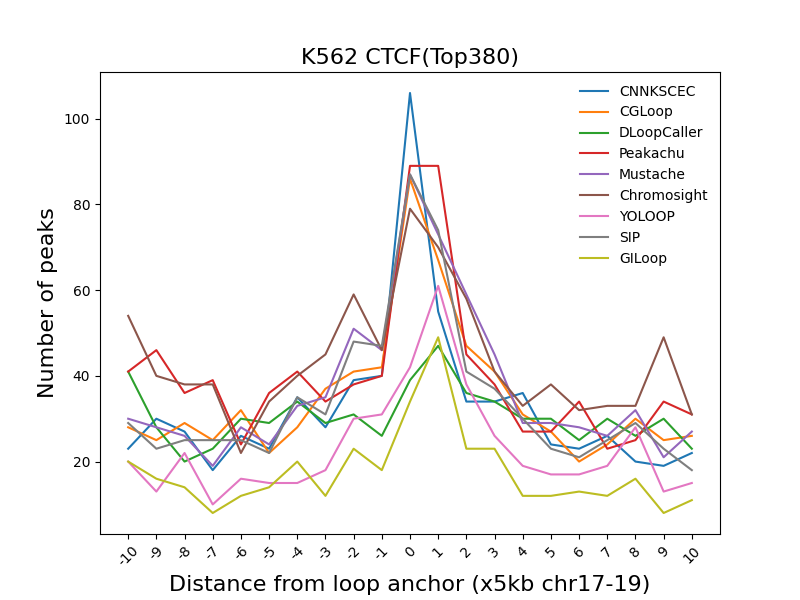

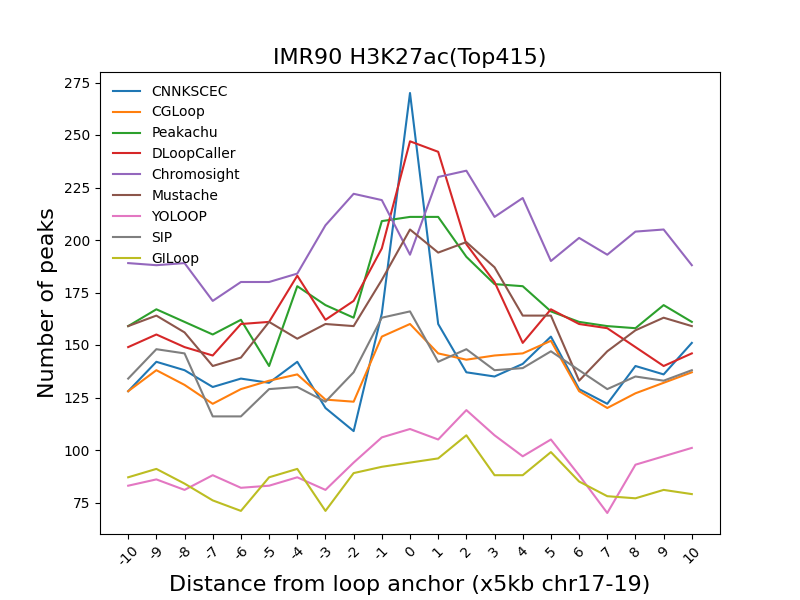

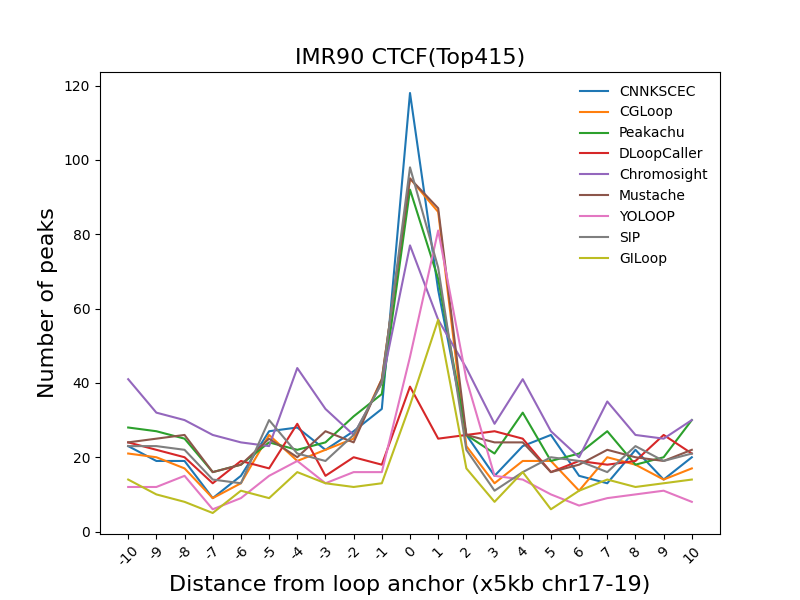

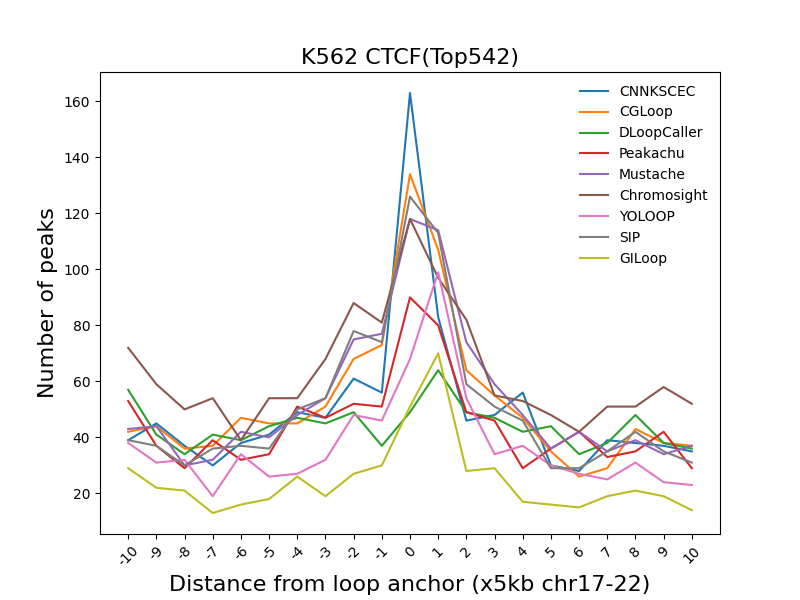

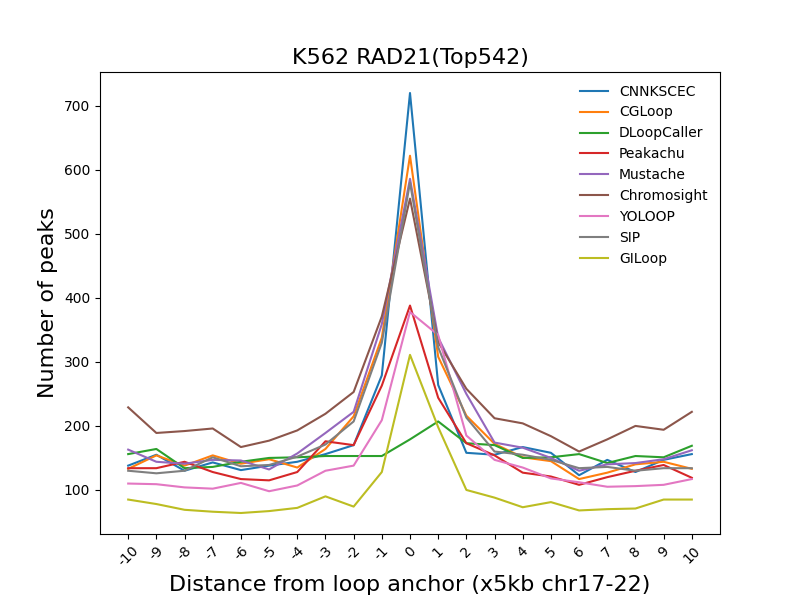

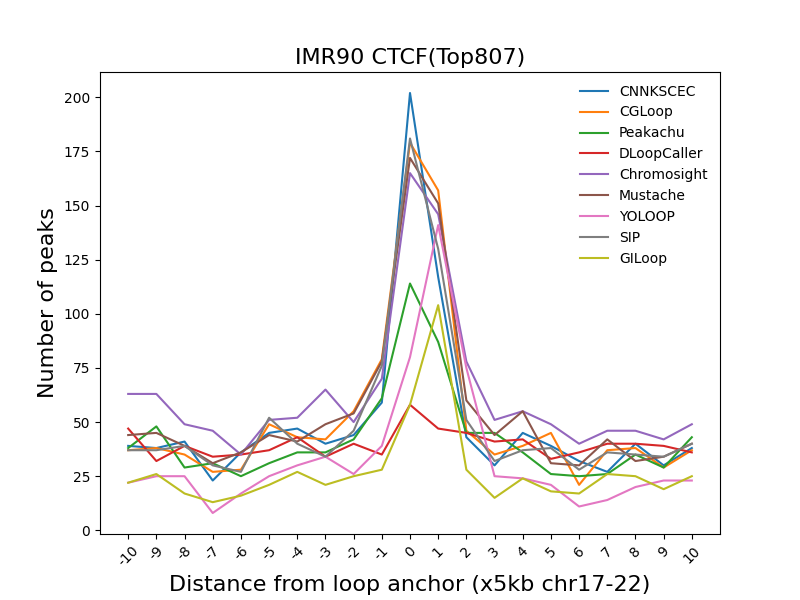

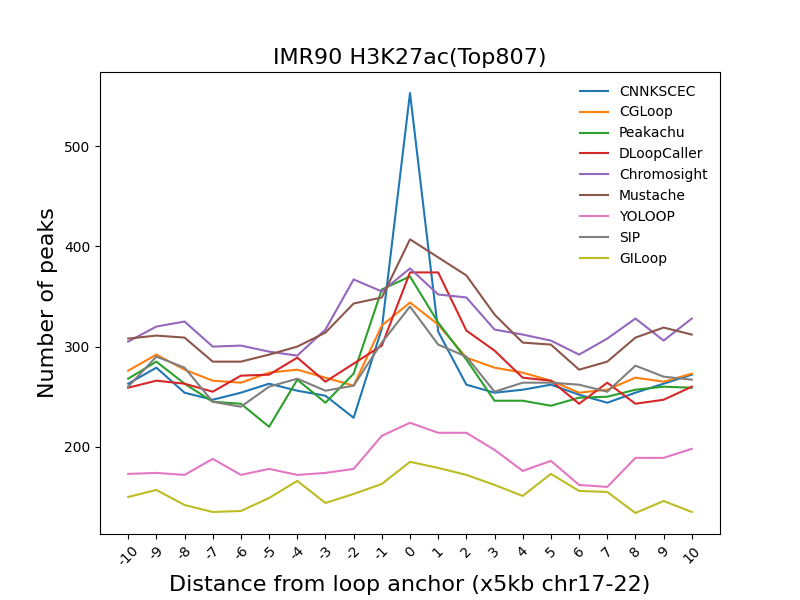


Figure S19. Protein factor peak analysis of predicted chromatin loops under a unified loop-number standard in K562 and IMR90

Table S1. Statistical significance analysis of F1-score configurations at different layers

| Comparison | Metric | Three-layer | Compared model | 95% CI of diff | Raw p | Holm-adjusted p | Significant |
| --- | --- | --- | --- | --- | --- | --- | --- |
| Three-layer vs Single-layer | F1-score | 0.9000 ± 0.0017 | 0.8976 ± 0.0014 | [0.0006, 0.0041] | 0.0128 | 0.0383 | True |
| Three-layer vs Double-layer | F1-score | 0.9000 ± 0.0017 | 0.8998 ± 0.0015 | [-0.0012, 0.0015] | 0.7998 | 1.0000 | False |
| Three-layer vs Four-layer | F1-score | 0.9000 ± 0.0017 | 0.8997 ± 0.0016 | [-0.0011, 0.0017] | 0.6540 | 1.0000 | False |

Table S2. Statistical significance analysis of PR-AUC values configured at different layers

| Comparison | Metric | Three-layer | Compared model | 95% CI of diff | Raw p | Holm-adjusted p | Significant |
| --- | --- | --- | --- | --- | --- | --- | --- |
| Three-layer vs Single-layer | PR-AUC | 0.9155 ± 0.0018 | 0.9134 ± 0.0012 | [0.0005, 0.0038] | 0.0170 | 0.0509 | False |
| Three-layer vs Double-layer | PR-AUC | 0.9155 ± 0.0018 | 0.9155 ± 0.0013 | [-0.0015, 0.0015] | 0.9828 | 1.0000 | False |
| Three-layer vs Four-layer | PR-AUC | 0.9155 ± 0.0018 | 0.9152 ± 0.0010 | [-0.0012, 0.0017] | 1.0000 | 1.0000 | False |

Table S3. Statistical significance analysis of the input data

| Metric | Hi-C+DNase mean±SD | Hi-C-only mean±SD | Mean difference | 95% CI of difference | Paired t-test p-value | Holm-adjusted p-value | Significant |
| --- | --- | --- | --- | --- | --- | --- | --- |
| F1-score | 0.9000 ± 0.0016 | 0.8739 ± 0.0023 | 0.0262 | [0.0246, 0.0277] | 3.479e^-11^ | 6.959e^-11^ | YES |
| PR-AUC | 0.9154 ± 0.0019 | 0.8944 ± 0.0021 | 0.0210 | [0.0190, 0.0230] | 1.883e^-09^ | 1.883e^-09^ | YES |

Table S4. Statistical significance analysis of F1-scores across different models

| comparison | Metric | CNNKSCEC | Compared model | Mean diff | 95% CI of diff | Raw p | Holm-adjusted p |
| --- | --- | --- | --- | --- | --- | --- | --- |
| CNNKSCEC vs teacher-only | F1-score | 0.9000 ± 0.0016 | 0.9006 ± 0.0018 | -0.0005 | [-0.0023, 0.0012] | 0.5025 | 0.5025 |
| CNNKSCEC vs student-only | F1-score | 0.9000 ± 0.0016 | 0.9004 ± 0.0011 | -0.0004 | [-0.0017, 0.0009] | 0.1309 | 0.2617 |

Table S5. Statistical significance analysis of PR-AUC across different models

| comparison | Metric | CNNKSCEC | Compared model | Mean diff | 95% CI of diff | Raw p | Holm-adjusted p |
| --- | --- | --- | --- | --- | --- | --- | --- |
| CNNKSCEC vs teacher-only | PR-AUC | 0.9154 ± 0.0019 | 0.9160 ± 0.0014 | -0.0006 | [-0.0024, 0.0011] | 0.4465 | 0.4465 |
| CNNKSCEC vs student-only | PR-AUC | 0.9154 ± 0.0019 | 0.9168 ± 0.0014 | -0.0015 | [-0.0030, 0.0001] | 0.0671 | 0.1342 |

# Availability of data and material

The download link for GM12878 cell line data is as follows:

Hi-C( Source: GEO, Identifier: GSE63525)：

<https://www.ncbi.nlm.nih.gov/geo/query/acc.cgi?acc=GSE63525>

DNase-seq( Source: ENCODE, Identifier: ENCFF264NMW):

<https://www.encodeproject.org/files/ENCFF264NMW/>

CTCF ChIA-PET( Source: Reference (Tang et al., 2015), Identifier: Tang, Z. et al. (2015)):

<https://doi.org/10.1016/j.cell.2015.11.024>

RAD21 ChIA-PET( Source: Reference (Heidari et al., 2014), Identifier: Heidari et al. (2014))：

<https://doi.org/10.1101/gr.176586.114>

H3k27ac HiChIP( Source: Reference (Mumbach et al., 2017), Identifier: Mumbach et al. (2017))：

<https://doi.org/10.1038/ng.3963>

SMC1 HiCHIP( Source: Reference (Mumbach et al., 2016), Identifier: Mumbach et al. (2016))：

<https://doi.org/10.1038/nmeth.3999>

CTCF ChIP-Seq( Source: ENCODE, Identifier: ENCSR000DZN):

<https://www.encodeproject.org/experiments/ENCSR000DZN/>

SMC3 ChIP-Seq( Source: ENCODE, Identifier: ENCSR000DZP):

<https://www.encodeproject.org/experiments/ENCSR000DZP/>

H3K27ac ChIP-Seq(bigwig)( Source: ENCODE, Identifier: ENCFF180LKW):

<https://www.encodeproject.org/files/ ENCFF180LKW/>

H3K4me1 ChIP-Seq(bigwig)( Source: ENCODE, Identifier: ENCFF682WPF):

<https://www.encodeproject.org/files/ENCFF682WPF/>

H3K4me3 ChIP-Seq(bigwig)( Source: ENCODE, Identifier: ENCFF674QZB):

<https://www.encodeproject.org/files/ENCFF674QZB/>

The download link for K562 cell line data is as follows:

Hi-C( Source: GEO, Identifier: GSE63525)：

<https://www.ncbi.nlm.nih.gov/geo/query/acc.cgi?acc=GSE63525>

DNase-seq( Source: ENCODE, Identifier: ENCFF352SET):

<https://www.encodeproject.org/files/ENCFF352SET/>

CTCF ChIA-PET( Source: ENCODE, Identifier: ENCFF001THV )：

<https://www.encodeproject.org/files/ENCFF001THV/>

RAD21 ChIA-PET( Source: ENCODE, Identifier: ENCFF002ENT):

<https://www.encodeproject.org/files/ENCFF002ENT/>

CTCF ChIP-Seq( Source: ENCODE, Identifier: ENCFF001XSU)：

<https://www.encodeproject.org/files/ENCFF001XSU/>

The download link for IMR90 cell line data is as follows:

Hi-C( Source:GEO, Identifier:GSE63525)：

<https://www.ncbi.nlm.nih.gov/geo/query/acc.cgi?acc=GSE63525>

DNase-seq( Source: ENCODE, Identifier: ENCFF291DOH):

<https://www.encodeproject.org/files/ENCFF291DOH/>

CTCF ChIP-PET( Source: ENCODE, Identifier: ENCFF464KWY)：

<https://www.encodeproject.org/files/ENCFF464KWY/>

CTCF ChIP-Seq( Source: ENCODE, Identifier:ENCFF001XSU):

<https://www.encodeproject.org/files/ENCFF001XSU/>

RAD21 ChIA-Seq( Source: ENCODE, Identifier: ENCSR000EFJ):

<https://www.encodeproject.org/experiments/ENCSR000EFJ/>

H3k27ac ChIP-Seq( Source: ENCODE, Identifier: ENCSR002YRE)：

<https://www.encodeproject.org/experiments/ENCSR002YRE/>
